# Structural Optimization and Improving Antitumor Potential of Moreollic Acid from *Gamboge*

**DOI:** 10.3390/molecules27020482

**Published:** 2022-01-13

**Authors:** Li-Zhi Cheng, Dan-Ling Huang, Min Liao, Ke-Ming Li, Zhao-Qiu Wu, Yong-Xian Cheng

**Affiliations:** 1 State Key Laboratory of Natural Medicines, School of Biopharmacy, China Pharmaceutical University, Nanjing 211198, China; lizhic18@gmail.com; 2 School of Pharmaceutical Sciences, Shenzhen University Health Science Center, Shenzhen 518060, China; 1910245009@email.szu.edu.cn (M.L.); kmli@szu.edu.cn (K.-M.L.); 3 Institute for Inheritance-Based Innovation of Chinese Medicine, Marshall Laboratory of Biomedical Engineering, School of Pharmaceutical Sciences, Shenzhen University Health Science Center, Shenzhen 518060, China

**Keywords:** *Gamboge*, structural optimization, synthesis, antitumor

## Abstract

Moreollic acid, a caged-tetraprenylated xanthone from *Gamboge*, has been indicated as a potent antitumor molecule. In the present study, a series of moreollic acid derivatives with novel structures were designed and synthesized, and their antitumor activities were determined in multifarious cell lines. The preliminary screening results showed that all synthesized compounds selectively inhibited human colon cancer cell proliferation. **TH12-10**, with an IC_50_ of 0.83, 1.10, and 0.79 μM against HCT116, DLD1, and SW620, respectively, was selected for further antitumor mechanism studies. Results revealed that **TH12-10** effectively inhibited cell proliferation by blocking cell-cycle progression from G1 to S. Besides, the apparent structure–activity relationships of target compounds were discussed. To summarize, a series of moreollic acid derivatives were discovered to possess satisfactory antitumor potentials. Among them, **TH12-10** displays the highest antitumor activities against human colon cancer cells, in which the IC_50_ values in DLD1 and SW620 are lower than that of 5-fluorouracil.

## 1. Introduction

Undoubtedly, cancer has become one of the severe diseases that threatens human health and imposes tremendous physical, emotional, and financial stress, and even death, on patients [1,2]. Nowadays, chemotherapeutics remains one of the major strategies for the treatment of cancer. Although plenty of chemotherapy antineoplastic drugs have been discovered, inevitable problems such as selectivity, resistance, and toxicity still linger [3,4]. Thus, seeking novel chemical agents with anticancer features is an urgent mission.

**Figure 1:**
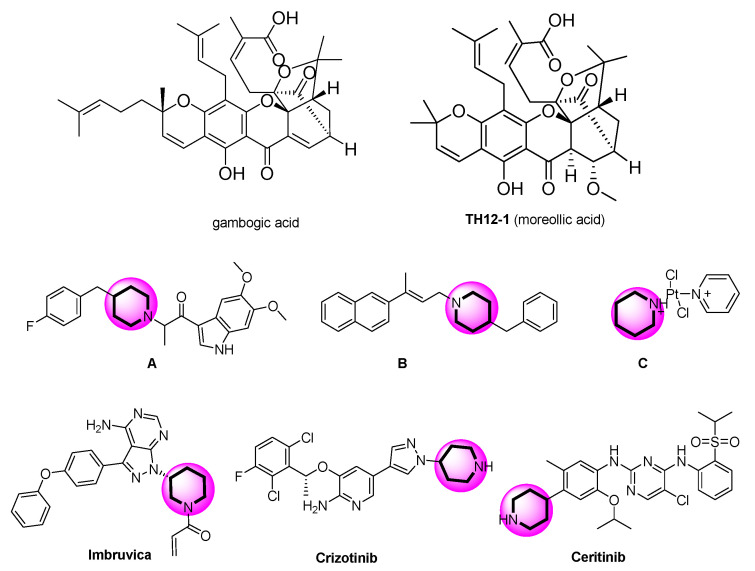
The structures of gambogic acid, moreollic acid, and active compounds containing the piperidine ring.

In recent years, we have focused on chemical and pharmacological investigations of natural products, and further uncovered a number of interesting molecules with satisfied antitumor activities [5,6,7,8]. *Gamboge,* the resin secreted by the *Garcinia hanburyi* tree, has been used as a natural health product in China for thousands of years [9]. *Gamboge* is also an effective remedy for treating inflammation, anabrosis, traumatic injury, parasites, tumor, and so on in clinics and folk medicine [10]. A mass of chemical and pharmacological research about *gamboge* has been carried out, and further indicated that the major components of *gamboge* are caged xanthones. In particular, gambogic acid (Figure 1) is the most representative caged xanthone in *gamboge*, and it displays antitumor and anti-proliferative effects, as well as antimicrobial activities [11,12,13]. It is reported that gambogic acid indeed inhibited the growth of various cancers, such as prostate cancer, lung cancer, gastric cancer, pancreatic cancer, leukemia, and hepato-carcinomatous [14,15,16,17]. Moreollic acid (**TH12-1**, Figure 1), an analogue of gambogic acid, was first isolated from *gamboge* by Yoshii’s group and it was demonstrated that it reveals cytotoxicity against cervical cancer cells [18]. However, there are fewer reports about further pharmacological and chemical studies of moreollic acid. At present, some problems such as promoting antitumor selectivity, enhancing effectiveness, and elucidating the antitumor mechanism of moreollic acid remain to be solved. On the other hand, the piperidine scaffold played a prominent role in the drug design, as it contributed to enhancing the interaction between the drug and the target. For example, Ferro et al. [19] developed the 4-(4-fluorine phenyl) piperidine fragment as a tyrosinase inhibitor to protect against melanoma, in which the piperidine ring of the active molecule (compound **A**, Figure 1) interacted with His263 via the p–p interaction, thereby promoting its bioactivity. Rui et al. [20] explored a small molecule containing the piperidine fragment as a sigma receptor (compound **B**, Figure 1), in which the piperidine portion played a key role in ligand binding to the receptor subtype and showed intensive adhesion towards the S2R receptor. In addition, the introduction of piperidine fragments into drug molecules is a common strategy for promoting pharmacokinetics. The classical instance is that the solubility of the cis-platinum derivative (compound **C**, Figure 1) improved remarkably due to decoration by the piperidine ring [21]. Significantly, many antitumor drugs approved by the FDA contain piperidine rings, i.e., Imbruvica, Crizotinib, Ceritinib, and so on [21]. Hence, we considered introducing the piperidine fragment (or its isosteres) into moreollic acid and expected to achieve a molecule with potential antitumor activity.

**Figure 2:**
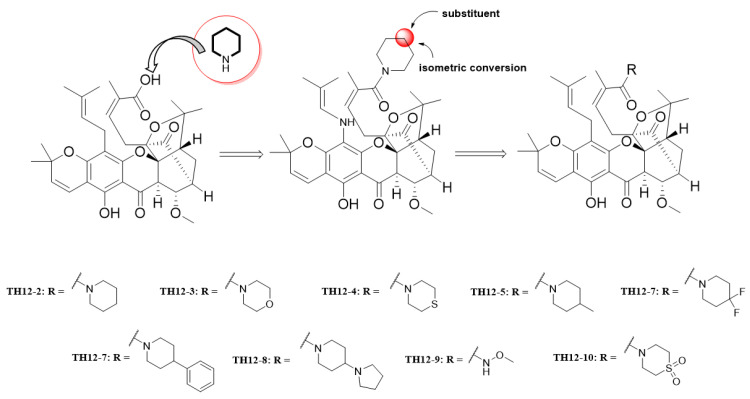
Design strategy of title compounds.

Inspired by the above-mentioned discussion, we initially isolated moreollic acid from *gamboge*, and then designed and synthesized 9 moreollic acid derivatives (**TH12-2**~**TH12-10**, Figure 2) via the decoration of the carboxyl to various amides bearing piperidine and the piperidine analogue. Subsequently, to go into more detail on the antitumor activities of these compounds, the cytotoxicity in human non-small-cell lung cancer cells (A549), human breast cancer cells (MDA-MB-231), human esophageal cancer cells (KYSE30), and human colon cancer cells (HCT-116, SW620, DLD1) was assayed. The results indicated that the target compounds displayed stronger cytotoxicity against human colon cancer cell lines than other tested cancer cell types. According to the IC_50_ value of the target compounds, **TH12-10** with the highest antiproliferation ability against human colon cancer cells was selected for subsequent evaluations of anticancer performance, as well as the superficial mechanism. It was revealed that **TH12-10** inhibits tumor cell proliferation by blocking cell-cycle progression from the G1 to the S phase. In particular, to our delight, these compounds displayed lower cytotoxicity against NCM460 cells (normal human colonic epithelial cells) than that of HCT-116, SW620, and DLD1.

As mentioned above, low selectivity and high toxicity are both the fatal defects of present antineoplastic drugs in the clinic. Here, we found a compound, **TH12-10**, with selective antitumor activity against colon cancer cells, which displayed more selectivity than the clinical drug 5-fluorouracil (5-Fu). Overall, our study provides a potential lead compound for the design of antineoplastic drugs for colon cancer.

## 2. Results and Discussion

### 2.1. Chemistry

**Scheme 1:**
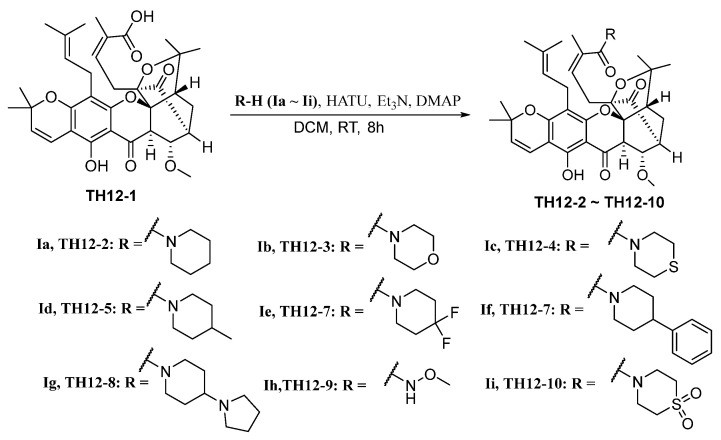
The synthesis pathway of compounds **TH12-2**~**TH12-10**.

Initially, compound **TH12-1** (moreollic acid) was isolated from *gamboge* as a yellowish solid. Its molecular formula was deduced as C_34_H_40_O_9_ by analysis of its positive high-resolution electrospray ionization mass spectroscopy (HRESIMS), nuclear magnetic resonance (NMR) (see Supplementary Materials). The accurate structure was confirmed by comparing the spectroscopic data with those reported in the literature [18]. As shown in Scheme 1, there was a further condensation reaction between **TH12-1** and corresponding amines (**Ia–Ii**), respectively, in the presence of 2-(7-Azabenzotriazol-1-yl)-N,N,N’,N’-tetramethyluronium hexafluorophosphate (HATU) to afford **TH12-2**~**TH12-10**.

### 2.2. Inhibition of Cell Viability in Tumor Cell Lines

Initially, the target compounds were evaluated for antiproliferative activities against human non-small-cell lung cancer cells (A549), human breast cancer cells (MDA-MB-231), human esophageal cancer cells (KYSE30), and human colon cancer cells (HCT-116) at 5 μM. All of them were tested by the CCK-8 assay using 5-Fu as the positive control. As shown in Table 1, the target compounds exhibited unequal inhibition activities against different tumor cells and showed more susceptibility to human colon cancer cells (HCT-116). Excitingly, most synthesized compounds possessed a higher inhibition ratio in HCT-116 cells than the positive control drug 5-Fu at 5 μM.

To further assess the antitumor activities of synthesized compounds against human colon cancer cells, the effects of each synthesized compound on the viability of two additional human colon cancer cells (SW620, DLD1) were evaluated, and normal human colonic epithelial cells (NCM460) were used as a control. The half-maximal inhibitory concentration (IC_50_) is shown in Table 2. It is obvious that all the synthesized compounds display potential antitumor activities against HCT116, DLD1, and SW620 cells. We observed that the cytotoxicity of most synthesized compounds was similar to 5-Fu (IC_50_ = 0.62 μM) in HTC116. In addition, for SW620, the activities of most optimized compounds were slightly stronger than that of 5-Fu. Moreover, for DLD1, all target compounds except **TH12-4** possess IC_50_ values ranging from 1.05 to 2.49 μM, which reveals more potent antitumor activities than 5-Fu (IC_50_ = 7.89 μM). Remarkably, **Th12-10** exhibited the most satisfactory antitumor activities (IC_50_ = 0.83, 1.10, and 0.79 μM in HCT116, DLD1, and SW620, respectively) among all target compounds, of which the anti-proliferation activity was close to that of 5-Fu against HCT116 and much better than that of 5-Fu against DLD1 and SW620 cells. It is worth mentioning that these compounds showed lower cytotoxicity against normal colonic epithelial cells than that of colon cancer cells. Taking **TH12-10** as an example, the IC_50_ values against HCT116, DLD1, and SW620 were 0.83, 1.10, and 0.79 μM, respectively, while the IC_50_ against NCM460 was 4.08 μM.

### 2.3. Apparent Structure–Activity Relationship

Structurally, the R group was a six-member nitrogenous heterocyclic skeleton with decorating in the 4-position. Based on the results of preliminary screening in human colon cancer cells, different substituent groups in the 4-position of R influence the antitumor activities of these compounds. Apparently, the incremental activity of the atom in the 4-position of R obeyed the rules as C > O > S, for which the cytotoxicity was correlated as follows: **TH12-2** > **TH12-3** > **TH12-4**. Interestingly, the antitumor activities of molecules improved visibly when S in the 4-position of R was oxidized to sulfone, as illustrated by the multiplied cytotoxicity of **Th12-10** (IC_50_ = 0.83, 1.10, and 0.79 μM in HCT116, DLD1, and SW620, respectively) compared to **Th12-4** (IC_50_ = 7.62, 7.89, and 7.26 μM in HCT116, DLD1, and SW620, respectively). Furthermore, when keeping the C atom as constant in the 4-position of R, the substituent in 4-C of R seems to provide a weak contribution to the antitumor activities against HCT116, DLD1, and SW620. For example, **TH12-2**, **TH12-5**, **TH12-6**, **TH12-7**, and **TH12-8** possess similar cytotoxicity levels.

### 2.4. ***TH12-10*** Effectively Inhibited the Cell Proliferation via Modulation of Cell-Cycle Regulatory Proteins

**Figure 3:**
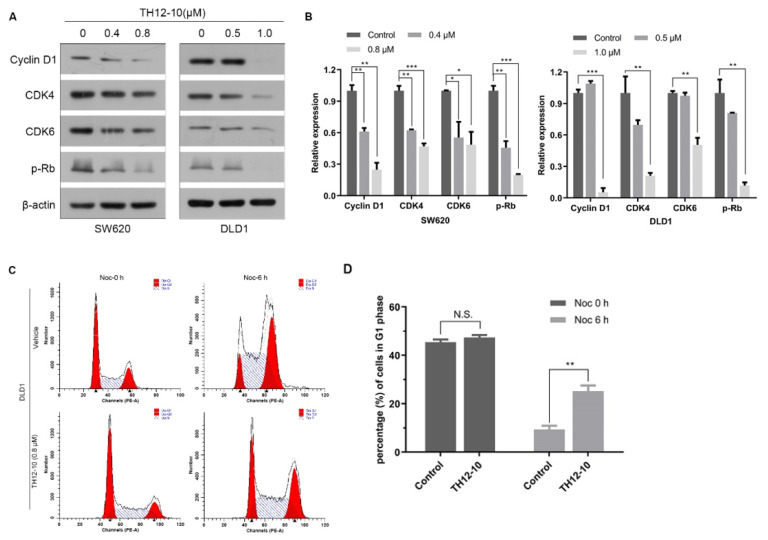
**TH12-10** blocks S phase entry. (**A**) Immunoblot analysis of Cyclin D1, CDK4, CDK6, and p-Rb in SW620 and DLD1 cells treated with indicated concentrations of **TH12-10** for 48 h. (**B**) Densitometry analysis of indicated proteins as described in (**A**) was performed using Image J software. (**C**) Flow cytometric analysis for cell-cycle distribution in DLD1 cells treated with **TH12-10** or vehicle and then with nocodazole for 48 h. (**D**) Quantitative analysis of DLD1 cells in G1 phase as described in (**C**). All data are presented as means ± SD of three independent experiments. * *p* < 0.05, ** *p* < 0.01, *** *p* < 0.001. N.S., not significant. Differences were tested using one-way ANOVA (**B**) or the *unpaired t-test* with Welch’s correction (**D**).

**Figure 4:**
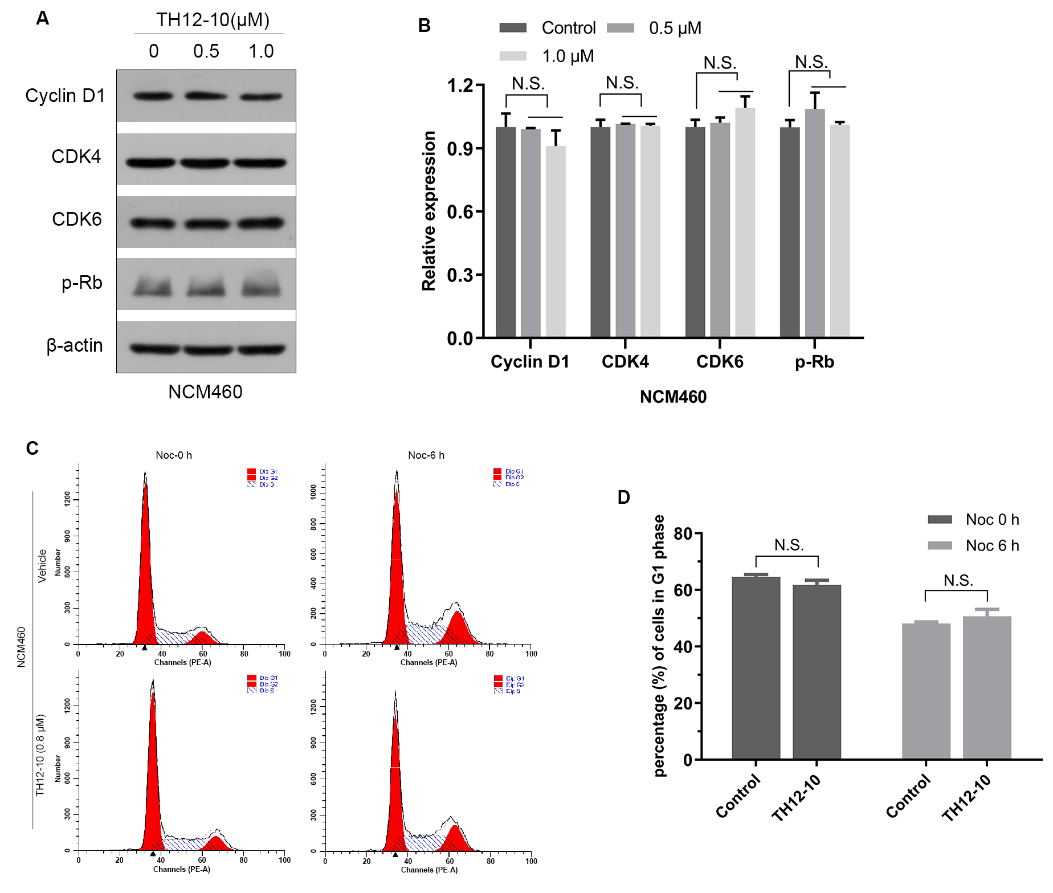
The contribution of **TH12-10** to the cell cycle. (**A**) Western blotting shows Cyclin D1, CDK4, CDK6, and p-Rb levels in NCM460 cells treated with **TH12-10** at indicated concentrations for 48 h. (**B**) Densitometry analysis of indicated proteins as described in (**A**) was conducted and presented. (**C**) Flow cytometric analysis for cell-cycle distribution in NCM460 cells treated with **TH12-10** or vehicle and then with nocodazole for a total of 48 h. (**D**) Quantitative analysis of NCM460 cells in G1 phase as described in (**C**). All data are presented as means ± SD of three independent experiments. N.S., not significant. Differences were tested using one-way ANOVA (**B**) or the *unpaired t-test* with Welch’s correction (**D**).

TH-12, one of the most potent anticancer compounds, was selected for further mechanism studies. To gain greater insight into whether the compound inhibits tumor cells’ proliferation via inducing tumor cell-cycle arrest, we performed protein analyses for cell-cycle-related proteins on SW620 and DLD1. As shown in Figure 3A,B, treating the SW620 and DLD1 cells with indicated concentrations of compound **Th12-10** for 48 h significantly decreased the expression of CyclinD1, cyclin-dependent kinase 6 (CDK6), cyclin-dependent kinase 4 (CDK4), and phosphorylated retinoblastoma (p-Rb), which are major components of the G1/S checkpoint. On the other hand, no significant change in the expression of related proteins was observed when treating the NCM460 cells with indicated concentrations of compound **Th12-10** (Figure 4A,B). Additionally, flow cytometric analyses were performed to determine cell-cycle distribution, where 400 ng/mL of nocodazole was used to synchronize cells in G2/M phase. Consistent with the immunoblotting data, 6 h after nocodazole administration, the percentage of DLD1 cells treated with TH12-10 in the G1 phase was significantly higher than that of blank control cells (Figure 3C,D), while the percentage of NCM460 cells treated with TH12-10 in the G1 phase was similar to that of blank control cells (Figure 4C,D). Collectively, the compound induced CDK6/CDK4 and CyclinD1 degradation, and subsequently decreased p-Rb levels, thereby blocking colon cancer cell-cycle progression from the G1 to the S phase [22].

## 3. Experimental Section

### 3.1. General Procedures

NMR spectra were recorded on a Bruker AV-500 and AV-600 spectrometer (Bruker, Karlsruhe, 3 Germany) with TMS as an internal standard. HRESIMS was collected by a Shimazu LC-20AD AB SCIEX triple TOF 5600+ MS spectrometer (Shimadzu Corporation, Tokyo, Japan). MCI gel CHP 20P (75–150 μm, Tokyo, Japan), C-18 silica gel (40–60 μm; Daiso Co., Tokyo, Japan), YMC gel ODS-A-HG (40–60 μm; YMC Co., Tokyo, Japan), silica gel (200–300 mesh; Qingdao Marine Chemical Inc., Qingdao, China), silica gel GF254 (80–100 mesh, Qingdao Marine Chemical Inc., China), and Sephadex LH-20 (Amersham Biosciences, Uppsala, Sweden) were used for column chromatography (CC). Semi-preparative HPLC was performed on a saipuruisi chromatograph with a Phenomenex Kinetex (250 × 10 mm, i.d., 5 μm) or a YMC-Pack ODS-A column (250 × 10 mm, i.d., 5 μm). Preparative HPLC was performed on a Chuangxin-Tongheng chromatograph equipped with a Thermo Hypersil GOLD-C_18_ column (250 × 21.2 mm, i.d., 5 μm). Racemic compounds and epimer were purified by chiral HPLC on a Daicel Chiralpak column (IC, 250 × 10 mm, i.d., 5 μm) and a Daicel Chiralpak column (IC, 250 × 4.6 mm, i.d., 5 μm).

### 3.2. Plant Resins

Garcinia resin was provided by Prof. Bin Qiu, Yunnan province, People’s Republic of China, in May 2018. The material was identified by Prof. Bin Qiu at Yunnan University of Traditional Chinese Medicine. A voucher specimen (CHYX0629) is deposited at the School of Pharmaceutical Sciences, Shenzhen University, People’s Republic of China.

### 3.3. Extraction and Isolation of ***TH12-1***

The resin (600 g) was extracted with 95% EtOH under the ultrasonic condition at room temperature three times (3 L × 1 h) and concentrated under reduced pressure to yield a crude extract. The extract (390 g) was separated on a silica gel column (200–300 mesh) eluted with gradient petroleum ether − acetone (100:0→50:50) to afford ten fractions (Fr.A–Fr.J). Fr.D (11.0 g) was subjected to Sephadex LH-20 (MeOH) to obtain two portions (Fr.D.1 and Fr.D.2). Subsequently, Fr.D.1 (10.5 g) was submitted to a silica gel column eluted with gradient petroleum ether−EtOH (100:0→100:20) to afford twelve fractions (Fr.D.1.1–Fr.D.1.12). Fr.D.1.9 (2.8 g) was subjected to Sephadex LH-20 (MeOH) to obtain two portions (Fr.D.1.9.1 and Fr.D.1.9.2). Fr.D.1.9.2 (1.5 g) was further purified by preparative HPLC (MeOH/H2O, 70%→100%, flow rate: 8 mL/min) to afford two portions (Fr.D.1.9.2.1 and Fr.D.1.9.2.2). Fr.D.1.9.2.1 (88.3 mg) was applied to semi-preparative HPLC (MeOH/H2O, containing 0.05% TFA, 95%) to obtain **TH12-1**, as well as moreollic acid (31.0 mg, tR = 20.4 min, flow rate: 3 mL/min).

Moreollic acid (**TH12-1**): yellow solid; ^1^H NMR (600 MHz, CDCl_3_) δ 11.93 (s, 1H), 6.61 (d, *J* = 10.0 Hz, 1H), 6.60–6.57 (m, 1H), 5.51 (d, *J* = 10.0 Hz, 1H), 5.02 (d, *J* = 1.5 Hz, 1H), 4.34 (dd, *J* = 4.6, 1.4 Hz, 1H), 3.31 (s, 3H), 3.30–3.25 (m, 1H), 3.23–3.15 (m, 3H), 3.07–3.11 (m, 1H), 2.84 (d, *J* = 1.5 Hz, 1H), 2.50 (d, *J* = 8.5 Hz, 1H), 1.97–1.94 (s, 3H), 1.96 (s, 3H), 1.74 (s, 3H), 1.63 (s, 3H), 1.46 (s, 3H), 1.41–1.38(m, 1H), 1.40 (s, 3H), 1.36 (s, 3H), 1.15 (s, 3H). ^13^C NMR (151 MHz, CDCl_3_) δ 208.5, 193.7, 171.4, 160.9, 155.6, 138.3, 131.3, 127.8, 126.4, 122.5, 115.3, 109.2, 103.1, 101.9, 88.4, 86.4, 82.4, 78.5, 74.0, 55.9, 47.9, 43.9, 43.5, 29.7, 28.6, 28.2, 27.9, 27.2, 25.7, 21.5, 20.7, 19.9, 18.1.

### 3.4. Synthesis of ***TH12-2*** and Its Analogues (***TH12-3**~**TH12-10***)

To a solution of **TH12-1** (5 mg, 8.5 μmol), triethylamine (4.3 mg, 42.5 μmol), HATU (6.4 mg, 17 μmol), and 4-dimethylaminopyridine (DMAP, 2 mg, 17 μmol) in 2 mL of dichloromethane (DCM), piperidine (1.4 mg, 17 mmol) was added under 0–5 °C and stirred for 30 min. Then, the mixture was stirred at ambient temperature for 5 h. The mixture was poured into 2 mL of 1M HCl aqueous solution. The organic layer was washed twice with 2 mL of saturated sodium chloride solution and dried over Na_2_SO_4_ and evaporated. The residue was purified by chromatography on a silica gel column using petroleum ether and acetone (*v*/*v*: 8/1) as the eluents to afford **TH12-2** as a yellow powder (4.8 mg, 85% yield). **TH12-3**~**TH12-10** were synthesized by a similar method, with the exception of replacing corresponding amines instead of piperazine.

**TH12-2**: Yield 85%; yellow powder, m.p. 121–123 °C; ^1^H NMR (600 MHz, CDCl_3_) δ 6.61 11.96 (s, 1H), (d, *J* = 10.0 Hz, 1H), 5.77 (s, 1H), 5.50 (d, *J* = 10.0 Hz, 1H), 5.04 (s, 1H), 4.33 (dd, *J* = 4.5, 1.3 Hz, 1H), 3.69–3.56 (m, 1H), 3.55–3.47 (m, 1H), 3.47–3.39 (m, 1H), 3.38–3.33 (m, 1H), 3.33–3.27 (m, 1H), 3.31 (s, 3H), 3.19–3.14 (m, 1H), 2.81–2.76 (m, 1H), 2.65 (t, *J* = 6.7 Hz, 2H), 2.46 (d, *J* = 8.5 Hz, 1H), 1.93 (dd, *J* = 14.6, 6.2 Hz, 1H), 1.87 (s, 3H), 1.76 (s, 3H), 1.66 (s, 3H), 1.54–1.52 (m, 3H), 1.44 (s, 3H), 1.39 (s, 3H), 1.38–1.33 (m, 1H), 1.31 (s, 3H), 1.11 (s, 3H), 0.91–0.78 (m, 4H). ^13^C NMR (150 MHz, CDCl_3_) δ 209.3, 194.1, 170.2, 161.0, 156.4, 155.8, 130.8, 129.1, 128.9, 126.1, 122.9, 115.4, 108.9, 102.9, 101.9, 88.2, 86.2, 81.7, 78.3, 74.2, 55.8, 47.3, 47.2, 44.4, 43.5, 41.8, 29.8, 29.7, 28.5, 28.2, 27.9, 27.3, 26.8, 25.6, 24.6, 21.6, 20.8, 20.0, 18.1. HRMS *m*/*z* calcd for C_39_H50NO_8_ (M + H)^+^ 660.3536; Found 660.3536.

**TH12-3:** Yield 77%; colorless oil; ^1^H NMR (600 MHz, CDCl_3_) δ 11.93 (s, 1H), 6.62 (d, *J* = 10.0 Hz, 1H), 5.90–5.82 (m, 1H), 5.51 (d, *J* = 10.0 Hz, 1H), 5.09–4.99 (m, 1H), 4.33 (dd, *J* = 4.5, 1.3 Hz, 1H), 3.69 (dd, *J* = 5.5, 3.3 Hz, 2H), 3.67–3.62 (m, 2H), 3.58–3.49 (m, 2H), 3.46–3.39 (m, 1H), 3.32 (s, 3H), 3.22 (s, 1H), 3.16 (dd, *J* = 14.5, 5.1 Hz, 1H), 2.80 (m, 1H), 2.68–2.62 (m, 2H), 2.47 (d, *J* = 8.5 Hz, 1H), 1.94 (dd, *J* = 14.6, 6.2 Hz, 1H), 1.88 (s, 3H), 1.76 (s, 3H), 1.66 (s, 3H), 1.45 (s, 3H), 1.39 (s, 3H), 1.31 (s, 3H), 1.30–1.24 (m, 2H), 1.11 (s, 3H). ^13^C NMR (150 MHz, CDCl_3_) δ 209.1, 193.8, 170.5, 161.0, 156.4, 155.7, 132.3, 131.1, 126.3, 123.8, 122.7, 115.3, 108.9, 103.0, 101.8, 88.3, 86.1, 81.8, 78.4, 74.2, 67.2, 66.8, 55.9, 47.7, 46.5, 44.2, 43.4, 41.3, 31.5, 30.1, 29.9, 28.6, 28.2, 27.3, 25.6, 21.6, 20.6, 20.0, 18.1. HRMS *m*/*z* calcd for C_38_H_48_NO_9_ (M + H)^+^ 662.3329; Found 662.3327.

**TH12-4:** Yield 78%; yellow oil; ^1^H NMR (600 MHz, CDCl_3_) δ 11.93 (s, 1H), 6.61 (d, *J* = 10.0 Hz, 1H), 5.84 (s, 1H), 5.50 (d, *J* = 10.0 Hz, 1H), 5.12–4.97 (m, 1H), 4.33 (dd, *J* = 4.5, 1.3 Hz, 1H), 3.98 (s, 1H), 3.83–3.74 (m, 2H), 3.69–3.61 (m, 1H), 3.33 (s, 3H), 3.30 (dd, *J* = 14.6, 8.3 Hz, 1H), 3.23 (s, 1H), 3.17 (dd, *J* = 14.5, 5.4 Hz, 1H), 3.11 (q, *J* = 7.3 Hz, 1H), 2.82–2.77 (m, 1H), 2.68–2.65 (m, 1H), 2.61–2.59 (m, 2H), 2.58–2.52 (m, 1H), 2.47 (d, *J* = 8.5 Hz, 1H), 1.94 (dd, *J* = 14.6, 6.2 Hz, 1H), 1.88 (s, 3H), 1.76 (s, 3H), 1.66 (s, 3H), 1.45 (s, 3H), 1.39 (s, 3H), 1.31 (s, 3H), 1.25 (t, *J* = 3.5 Hz, 2H), 1.11 (s, 3H). ^13^C NMR (150 MHz, CDCl_3_) δ 209.2, 193.9, 170.7, 161.0, 156.4, 155.7, 131.0, 126.2, 123.7, 122.7, 115.3, 108.9, 103.0, 101.8, 88.2, 86.1, 81.8, 78.4, 74.3, 56.0, 48.7, 47.6, 44.3, 43.4, 43.2, 35.0, 34.4, 31.5, 31.4, 30.3, 30.2, 30.1, 29.9, 29.7, 28.6, 28.4, 28.2, 28.0, 27.6, 27.3, 25.7, 20.8, 20.0, 18.1. HRMS *m*/*z* calcd for C_38_H_48_NO_8_S (M + H)^+^ 678.3101; Found 678.3099.

**TH12-5:** Yield 85%; yellow oil; ^1^H NMR (600 MHz, CDCl_3_) δ 11.96 (d, *J* = 7.5 Hz, 1H), 6.61 (d, *J* = 10.0 Hz, 1H), 5.77 (d, *J* = 6.8 Hz, 1H), 5.50 (d, *J* = 10.0 Hz, 1H), 5.04 (d, *J* = 6.8 Hz, 1H), 4.53 (t, *J* = 14.7 Hz, 1H), 4.33 (d, *J* = 4.5 Hz, 1H), 3.87–3.78 (m, 1H), 3.32–3.28 (m, 1H), 3.30 (s, 3H), 3.16 (d, *J* = 14.7 Hz, 1H), 3.01–2.89 (m, 1H), 2.80–2.77 (m, 1H), 2.71–2.55 (m, 3H), 2.46 (dd, *J* = 8.5, 1.9 Hz, 1H), 1.96–1.91 (m, 1H), 1.86 (s, 3H), 1.76 (s, 3H), 1.70–1.63 (m, 1H) 1.66 (s, 3H), 1.46–1.44 (m, 3H), 1.42 (s, 3H), 1.40–1.38 (m, 3H), 1.32–1.30 (m, 3H), 1.25 (s, 3H), 1.11 (s, 3H), 0.94 (d, *J* = 6.5 Hz, 3H). ^13^C NMR (150 MHz, CDCl_3_) δ 209.2, 194.0, 170.1, 160.9, 156.3, 155.8, 138.8, 130.8, 126.1, 123.4, 115.3, 108.9, 102.8, 101.8, 88.1, 86.2, 81.5, 78.3, 74.1, 55.7, 47.3, 44.3, 43.5, 41.1, 34.9, 34.4, 33.7, 31.4, 30.3, 30.1, 29.8, 27.2, 25.6, 21.6, 20.7, 20.0, 18.0. HRMS *m*/*z* calcd for C_40_H_52_NO_8_ (M + H)^+^ 674.3693; Found 674.3690.

**TH12-6:** Yield 91%; white powder, m.p. 94–96 °C; ^1^H NMR (600 MHz, CDCl_3_) δ 11.94 (s, 1H), 6.64 (d, *J* = 10.0 Hz, 1H), 5.98–5.87 (m, 1H), 5.53 (d, *J* = 10.0 Hz, 1H), 5.11–5.00 (m, 1H), 4.35 (dd, *J* = 4.5, 1.4 Hz, 1H), 3.99–3.90 (m, 1H), 3.76–3.67 (m, 1H), 3.61 (dt, *J* = 12.8, 6.2 Hz, 1H), 3.53 (ddd, *J* = 13.0, 8.3, 4.1 Hz, 1H), 3.36–3.30 (m, 1H), 3.33 (s, 3H), 3.21–3.16 (m, 1H), 2.85–2.79 (m, 1H), 2.74–2.66 (m, 2H), 2.61 (dd, *J* = 15.6, 8.8 Hz, 2H), 2.49 (d, *J* = 8.5 Hz, 1H), 2.09–1.93 (m, 4H), 1.92 (s, 4H), 1.78 (s, 4H), 1.69 (s, 4H), 1.48 (s, 3H), 1.47–1.43 (m, 1H), 1.42 (s, 3H), 1.40–1.35 (m, 1H), 1.33 (s, 3H), 1.32–1.25 (m, 2H), 1.13 (s, 3H). ^13^C NMR (150 MHz, CDCl_3_) δ 209.2, 193.7, 170.5, 160.9, 156.3, 155.6, 132.3, 131.1, 126.2, 124.0 (t, *J* = 345.3 Hz), 122.5, 120.1, 115.2, 108.9, 103.0, 101.7, 88.3, 85.9, 81.8, 78.4, 74.2, 55.9, 47.7, 44.1, 43.3, 42.89 (t, *J* = 4.8 Hz), 37.60 (t, *J* = 5.1 Hz), 34.60 (t, *J* = 23.0 Hz), 33.66 (t, *J* = 23.1 Hz), 29.8, 28.5, 28.2, 27.9, 27.2, 25.6, 21.5, 20.6, 19.9, 18.0. HRMS *m*/*z* calcd for C_39_H_48_NO_8_F_2_ (M + H)^+^ 696.3348; Found 696.3347.

**TH12-7:** Yield 85%; yellow powder, 115–116 °C; ^1^H NMR (600 MHz, CDCl_3_) δ 11.95 (d, *J* = 11.2 Hz, 1H), 7.30 (t, *J* = 7.6 Hz, 2H), 7.19 (dd, *J* = 18.3, 7.4 Hz, 3H), 6.61 (d, *J* = 10.0 Hz, 1H), 5.83 (d, *J* = 27.9 Hz, 1H), 5.50 (d, *J* = 10.0 Hz, 1H), 5.08–5.08 (m, 1H), 4.81–4.70 (m, 1H), 4.02 (dd, *J* = 55.5, 13.5 Hz, 1H), 3.35–3.30 (m, 2H), 3.29 (s, 3H), 3.24–3.01 (m, 2H), 2.80 (dt, *J* = 16.0, 5.3 Hz, 1H), 2.75–2.62 (m, 3H), 2.50–2.43 (m, 1H), 1.91 (s, 3H), 1.95–1.84 (m, 4H), 1.78 (s, 3H), 1.67 (s, 3H), 1.66–1.61 (m, 2H), 1.45 (s, 3H), 1.39 (s, 3H), 1.32 (s, 3H), 1.12 (s, 3H). ^13^C NMR (150 MHz, CDCl_3_) δ 209.1, 194.1, 170.4, 161.0, 156.4, 155.8, 145.4, 145.2, 135.8, 133.3, 131.0, 130.9, 128.5, 128.5, 126.8, 126.7, 126.2, 123.2, 122.9, 115.4, 108.9, 103.0, 101.9, 86.2, 81.8, 81.6, 78.3, 77.2, 77.0, 76.8, 74.2, 55.8, 47.4, 46.9, 43.5, 43.0, 42.8, 41.5, 33.2, 32.9, 29.9, 28.6, 28.2, 28.1, 27.3, 25.7, 21.7, 21.6, 20.8, 20.0, 18.1. HRMS *m*/*z* calcd for C_45_H5_4_NO8 (M + H)^+^ 736.3849; Found 736.3846.

**TH12-8:** Yield 62%; yellow oil; ^1^H NMR (600 MHz, CDCl_3_) δ 11.90 (s, 1H), 6.62 (d, *J* = 10.0 Hz, 1H), 5.91 (d, *J* = 39.4 Hz, 1H), 5.52 (d, *J* = 10.0 Hz, 1H), 5.04 (brs, 1H), 4.88 (d, *J* = 13.9 Hz, 1H), 4.34 (d, *J* = 4.1 Hz, 1H), 4.20 (d, *J* = 12.2 Hz, 1H), 3.78–3.57 (m, 2H), 3.39–3.36 (m, 2H), 3.29 (s, 3H), 3.10–3.00 (m, 2H), 3.17–3.14 (m, 2H), 3.06–3.04 (m, 1H), 2.87 (brs, 1H), 2.79–2.77 (m, 2H), 2.67–2.57 (m, 1H), 2.51–2.46 (m, 1H), 2.14 –1.99 (m, 10H), 1.87 (s, 3H), 1.77 (s, 3H), 1.66 (s, 3H), 1.47 (s, 3H), 1.39 (s, 3H), 1.32–1.34 (m,2H), 1.28 (s, 3H), 1.06 (s, 3H). ^13^C NMR (150 MHz, CDCl_3_) δ 209.3, 193.8, 161.0, 156.4, 155.6, 133.4, 131.1, 126.3, 122.7, 122.1, 115.3, 109.0, 108.9, 103.1, 103.0, 101.9, 88.2, 86.2, 81.8, 78.4, 74.0, 61.8, 55.8, 51.6, 51.3, 47.2, 44.3, 43.4, 29.8, 28.6, 28.2, 27.9, 27.2, 25.7, 23.3, 23.2, 21.7, 21.6, 20.7, 20.0, 19.9, 18.1, 9.0. HRMS *m*/*z* calcd for C_43_H_57_ N_2_O_8_ (M + H)^+^ 729.4115; Found 729.4108.

**TH12-9:** Yield 90%; yellow oil; ^1^H NMR (600 MHz, CDCl_3_) δ 11.90 (s, 1H), 9.93 (s, 1H), 6.62 (d, *J* = 10.0 Hz, 1H), 6.00–5.87 (m, 1H), 5.53 (d, *J* = 10.0 Hz, 1H), 5.07–4.97 (m, 1H), 4.38 (dd, *J* = 4.6, 1.2 Hz, 1H), 3.87 (s, 3H), 3.34 (s, 3H), 3.33–3.26 (m, 1H), 3.18 (dd, *J* = 14.6, 5.7 Hz, 1H), 2.89–2.82 (m, 2H), 2.69 (dd, *J* = 15.0, 7.5 Hz, 1H), 2.53 (d, *J* = 8.5 Hz, 1H), 2.00–1.92 (m, 1H), 1.97 (s, 3H), 1.73 (s, 3H), 1.65 (s, 3H), 1.46 (s, 3H), 1.43–1.37 (m, 1H), 1.40 (s, 4H), 1.36 (s, 3H), 1.17 (s, 3H). ^13^C NMR (150 MHz, CDCl_3_) δ 208.5, 193.2, 166.6, 161.1, 156.4, 155.2, 133.0, 131.4, 126.5, 126.1, 122.4, 115.2, 109.3, 103.3, 101.8, 88.5, 86.7, 82.9, 78.6, 73.8, 64.3, 56.0, 48.3, 43.7, 43.2, 29.8, 28.6, 28.2, 27.7, 27.3, 25.7, 21.4, 20.8, 19.8, 18.1. HRMS *m*/*z* calcd for C_35_H_44_NO_9_ (M + H)^+^ 622.3016; Found 622.3014.

**TH12-10:** Yield 72%; yellow powder, 138–140 °C; ^1^H NMR (600 MHz, Chloroform-*d*) δ 11.88 (s, 1H), 6.62 (d, *J* = 10.0 Hz, 1H), 5.96 (d, *J* = 5.0 Hz, 1H), 5.52 (d, *J* = 10.0 Hz, 1H), 5.04 (s, 1H), 4.44 (brs, 1H), 4.33 (dd, *J* = 4.5, 1.2 Hz, 1H), 4.13 (d, *J* = 14.1 Hz, 1H), 3.94–3.80 (m, 1H), 3.72 (q, *J* = 7.0 Hz, 1H), 3.36–3.26 (m, 3H), 3.33 (s, 3H), 3.19–3.02 (m, 3H), 3.00–2.93 (m, 1H), 2.85–2.79 (m, 1H), 2.68 (d, *J* = 15.5 Hz, 1H), 2.55 (dd, *J* = 15.8, 9.7 Hz, 1H), 2.49 (d, *J* = 8.5 Hz, 1H), 1.97–1.92 (m, 1H), 1.92 (s, 3H), 1.76 (s, 3H), 1.67 (s, 3H), 1.46 (s, 3H), 1.40 (s, 3H), 1.39–1.35 (m, 2H), 1.32 (s, 3H), 1.25 (t, *J* = 7.0 Hz, 1H), 1.11 (s, 3H). ^13^C NMR (150 MHz, CDCl_3_) δ 209.3, 193.4, 170.9, 160.8, 156.3, 155.5, 131.6, 131.4, 126.3, 125.5, 122.3, 115.2, 108.9, 103.1, 101.7, 88.4, 85.8, 82.0, 78.5, 74.2, 56.1, 52.0, 51.6, 47.9, 44.5, 43.9, 43.2, 39.3, 29.9, 28.5, 28.1, 28.1, 27.2, 25.6, 21.6, 20.6, 19.9, 18.0. HRMS *m*/*z* calcd for C_38_H_48_NO_10_S (M + H)^+^ 710.2999; Found 710.2994.

### 3.5. Bioactivy Assay

#### 3.5.1. The Cell Culture

HCT116, SW620, and DLD1 were purchased from American Type Culture Collection (ATCC). SW620 and DLD1 were cultured in Dulbecco’s Modified Eagles medium (Thermo), and HCT116 was cultured in McCoy’s 5A medium (Thermo). All media were supplemented with 10% fetal bovine serum (Thermo) and 1% penicillin/streptomycin. All the cells were passaged at approximately 80% confluency. Cells were tested for mycoplasma every two months, and only mycoplasma-negative cells were used

#### 3.5.2. Cell Proliferation Assay

To investigate the effects of compounds on tumor cells’ proliferation, the cell proliferation assay was performed using the Cell Counting Kit-8 (#40203ES60, Yeasen, Shanghai, China). In brief, cells were seeded in a 96-well plate at 3 × 10^3^ cells per well and cultured overnight. Then, the medium was replaced with fresh DMEM medium containing various concentrations of compounds or vehicle. After 48 h of incubation, the medium was exchanged with 100 μL of FBS-free DMEM medium supplemented with 10 μL of CCK8 added to each well and incubated at 37 °C for another 2 h. Absorbance at 450 nm was measured with a microplate reader (Thermo). The experiments were performed in triplicate. The mean ± SD values presented in the figures were calculated from three independent experiments. Comparisons between groups were evaluated using GraphPad Prism version 7.0 software. *p* < 0.05 was considered statistically significant. 

#### 3.5.3. Immunoblot

When 80% confluency was reached, cells were seeded into a 6-well plate at 3 × 10^6^ cells per well and allowed to attach overnight. Then, the cells were treated with different concentrations of **TH12-10** or vehicle for 48 h. Cells were lysed in RIPA buffer (#89900, Thermo, Waltham, MA, USA) supplemented with protease inhibitor cocktail (#B14002, Bimake, Houston, TX, USA) on ice for 30 min and centrifuged at 12,000 rpm for 10 min at 4 °C. Protein concentration was determined by the BCA kit and equal amounts of lysates were separated by SDS-polyacrylamide gels, and transferred to nitrocellulose membranes (#HATF00010, Millipore, Burlington, MA, USA). Then, the membranes were blocked with 5% milk in TBST for 1 h and probed with the indicated antibodies. Antibodies used for the immunoblot analysis in this study were as follows: Cyclin D1 (55506T, Cell Signaling Technology, Danvers, MA, USA), CDK4 (ab199728, Abcam, Cambridge, UK), CDK6 (13331T, Cell Signaling Technology, Danvers, MA, USA), phospho-Rb (Ser807/811) (8516S, Cell Signaling Technology, Danvers, MA, USA), and β-actin (sc-47778, Santa Cruz Biotechnology, CA, USA)

## 4. Conclusions

A series of moreollic acid derivatives, with novel structures designed and synthesized, and their antitumor activities were evaluated. **TH12-10** showed the highest antitumor activity against DLD1 and SW620 cells, with IC_50_ values of 1.10 and 0.79 μM, respectively, superior to those of 5-Fu. In addition, further mechanism studies recommended that **TH12-10** effectively inhibited cell proliferation by blocking cell-cycle progression from G1 to S. In addition, the structure–activity relationship discussion suggested that substituent groups in the 4-position of R affect the bioactivities of title compounds. This work provided useful guidance for the optimization of antitumor molecules from the natural product *gamboge*.

## Figures and Tables

**Table 1:** Inhibition rates (%) of compounds **TH12-2**~**TH12-10** against human tumor cells at 5 μM.

Compound	R	5 μM			
		A549	MDA-MB-231	KYSE30	HCT-116
**TH12-1**	OH	18.38 ± 0.02	−4.9 ± 0.04	20.76 ± 0.04	83.86 ± 1.10 2.07
**TH12-2**	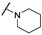	2.51 ± 4.32	0.14 ± 3.51	9.08 ± 2.44	86.08 ± 2.83
**TH12-3**	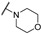	11.6 ± 4.25	1.71 ± 1.66	10.63 ± 2.66	87.36 ± 2.49
**TH12-4**	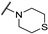	11.08 ± 5.41	3.13 ± 1.16	6.98 ± 1.99	16.27 ± 4.56
**TH12-5**	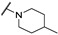	14.3 ± 2.84	0.69 ± 1.92	5.65 ± 3.77	85.48 ± 1.45
**TH12-6**	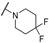	12.56 ± 0.71	35.5 ± 1.17	27.02 ± 3.21	87.23 ± 3.07
**TH12-7**	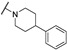	4.59 ± 1.27	2.99 ± 0.16	−2.71 ± 0.83	87.14 ± 2.37
**TH12-8**	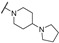	4.8 ± 0.63	4.32 ± 1.96	4.04 ± 1.50	87.57 ± 2.76
**TH12-9**	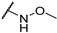	8.54 ± 1.11	−1.76 ± 0.43	4.49 ± 1.05	83.86 ± 2.07
**TH12-10**	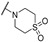	6.85 ± 2.16	8.09 ± 0.28	3.65 ± 2.44	88.09 ± 2.78
**5-Fu**		21.14 ± 1.79	26.59 ± 2.51	27.96 ± 2.27	74.45 ± 1.10

**Table 2:** IC_50_ values (μM) of **TH12-1**~**TH12-10** against HCT116, DLD1, and SW620.

Compound	R	IC_50_ ± SD (μM)			
		HCT116	DLD1	SW620	NCM460
**Th12-1**	OH	1.71 ± 0.33	1.44 ± 0.29	1.49 ± 0.37	3.16 ± 0.32
**Th12-2**	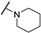	1.19 ± 0.25	1.39 ± 0.19	1.15 ± 0.13	4.73 ± 0.04
**Th12-3**	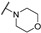	1.96 ± 0.20	2.308 ± 0.23	1.82 ± 0.33	4.72 ± 0.03
**Th12-4**	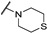	7.62 ± 0.93	7.89 ± 0.12	7.26 ± 0.87	19.9 ± 0.84
**Th12-5**	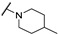	2.37 ± 0.63	2.49 ± 0.56	1.97 ± 0.34	5.11 ± 0.21
**Th12-6**	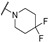	1.16 ± 0.23	1.22 ± 0.2	1.07 ± 0.16	2.49 ± 0.16
**Th12-7**	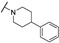	1.52 ± 0.14	1.45 ± 0.25	1.31 ± 0.24	2.97 ± 0.08
**Th12-8**	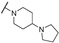	1.00 ± 0.13	1.05 ± 0.23	0.97 ± 0.10	2.29 ± 0.01
**Th12-9**	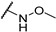	1.68 ± 0.26	1.67 ± 0.70	1.59 ± 0.44	1.63 ± 0.02
**Th12-10**	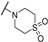	0.83 ± 0.10	1.10 ± 0.19	0.79 ± 0.12	4.08 ± 0.11
**5-Fu**		0.62 ± 0.02	7.89 ± 0.75	2.68 ± 0.01	13.59 ± 0.59

## Data Availability

All the datasets on which the conclusions of the manuscript rely are presented in the paper.

## Supplementary Materials

The following are available online at online, Figure S1–S30: the 1H NMR, 13C NMR and HRMS spectra of the compounds **TH12-1**~**TH12-10**.

## Sample Availability

Samples of the compounds **TH12-1**~**TH12-10** are available from the authors.
