# Supplementary material for: Structural Optimization and Improving Antitumor Potential of Moreollic Acid from Gamboge
Source: Molecules. 2022 Jan 13;27(2):482. doi: 10.3390/molecules27020482 (PMC8846360; doi:10.3390/molecules27020482)
Supplement: Supplementary file 1 [file molecules-27-00482-s001.zip › molecules-1507784-supplementary.pdf]

Supplementary Materials: Structural Optimization and Improving Antitumor Potential of Moreollic Acid from *Gamboge*

Li-Zhi Cheng [1,†], Danling Huang [2,3,†]*, Min Liao [2,3], Ke-Ming Li [2,3], Zhao-Qiu Wu [1]*, Yong-Xian Cheng[2,3]*

1 State Key Laboratory of Natural Medicines, School of Biopharmacy, China Pharmaceutical University, Nanjing 211198, China; lizhic18@gmail.com (L. -Z. Cheng); zqwu@cpu.edu.cn (Z. -Q. Wu)

2 Guangdong Key Laboratory for Genome Stability & Disease Prevention, School of Pharmaceutical Sciences, Shenzhen University Health Science Center, Shenzhen 518060, China; 1910245009@email.szu.edu.cn (M. Liao); kmli@szu.edu.cn (K.-M. Li); leonchemistry@szu.edu.cn (D. Huang); Yong-Xian Cheng@szu.edu.cn (Y.-X. Cheng.);

3 Institute for Inheritance-Based Innovation of Chinese Medicine, Marshall Laboratory of Biomedical Engineering, School of Pharmaceutical Sciences, Health Science Center, Shenzhen University, Shenzhen 518060, China

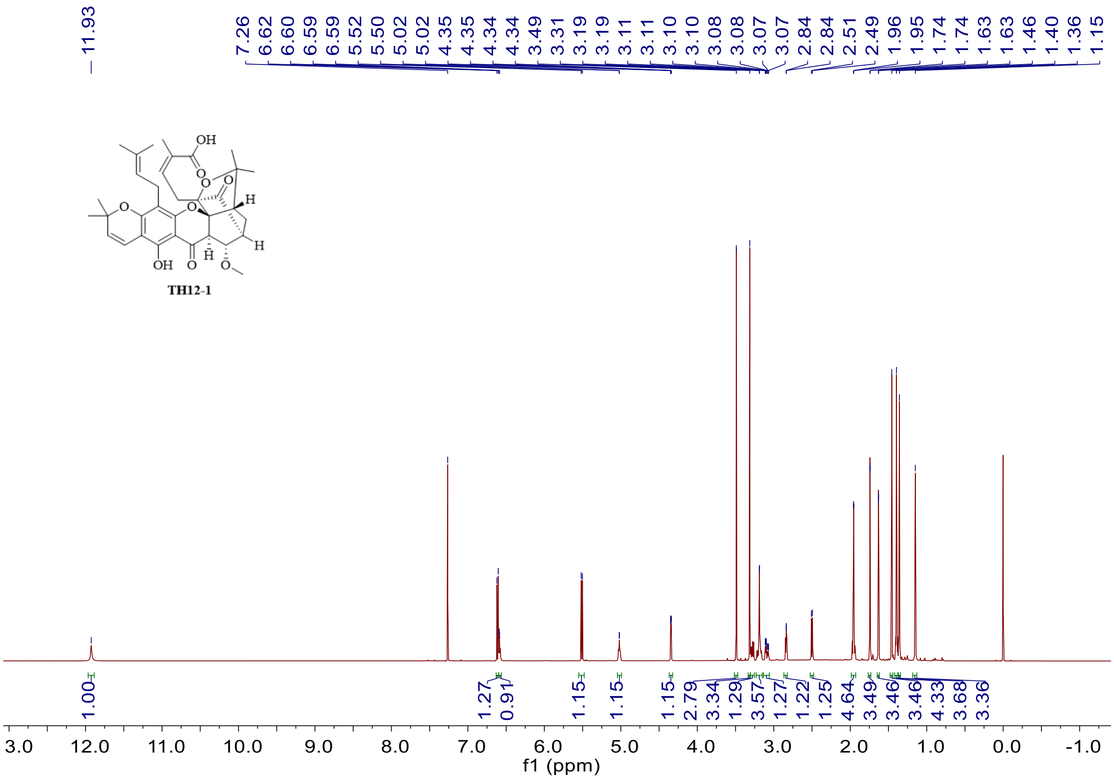

Figure S1. ^{1}HNMR of TH12-1

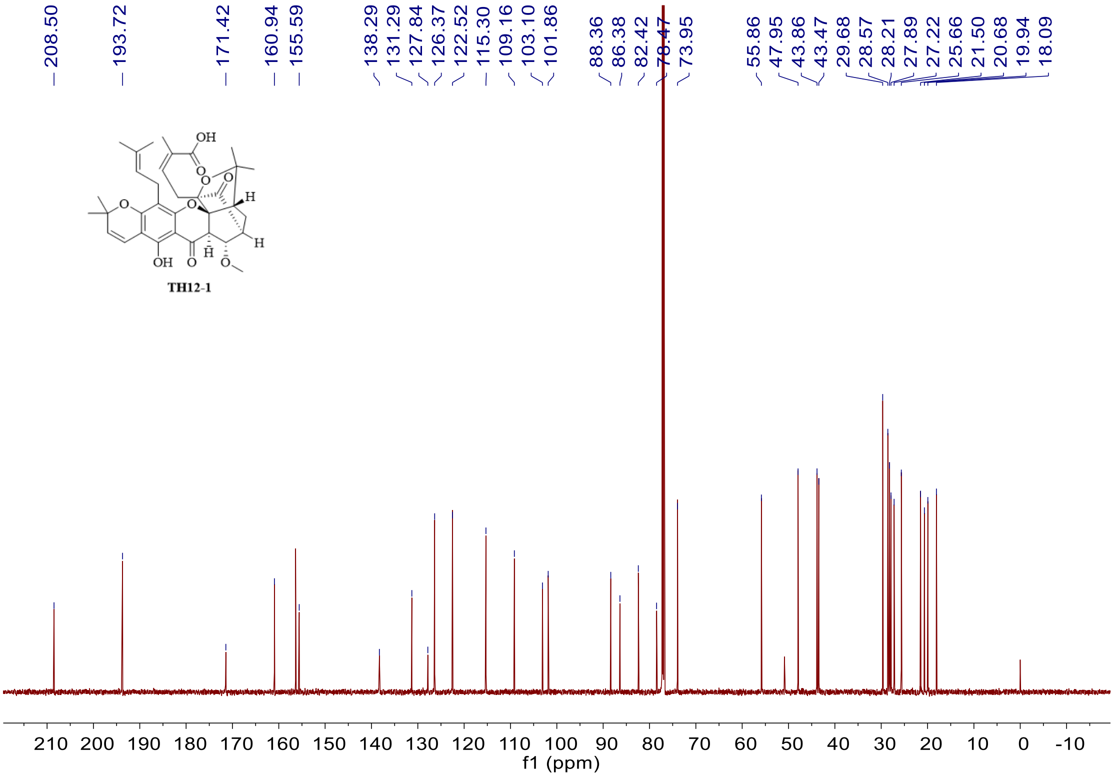

Figure S2. ^{13}C NMR of TH12-1

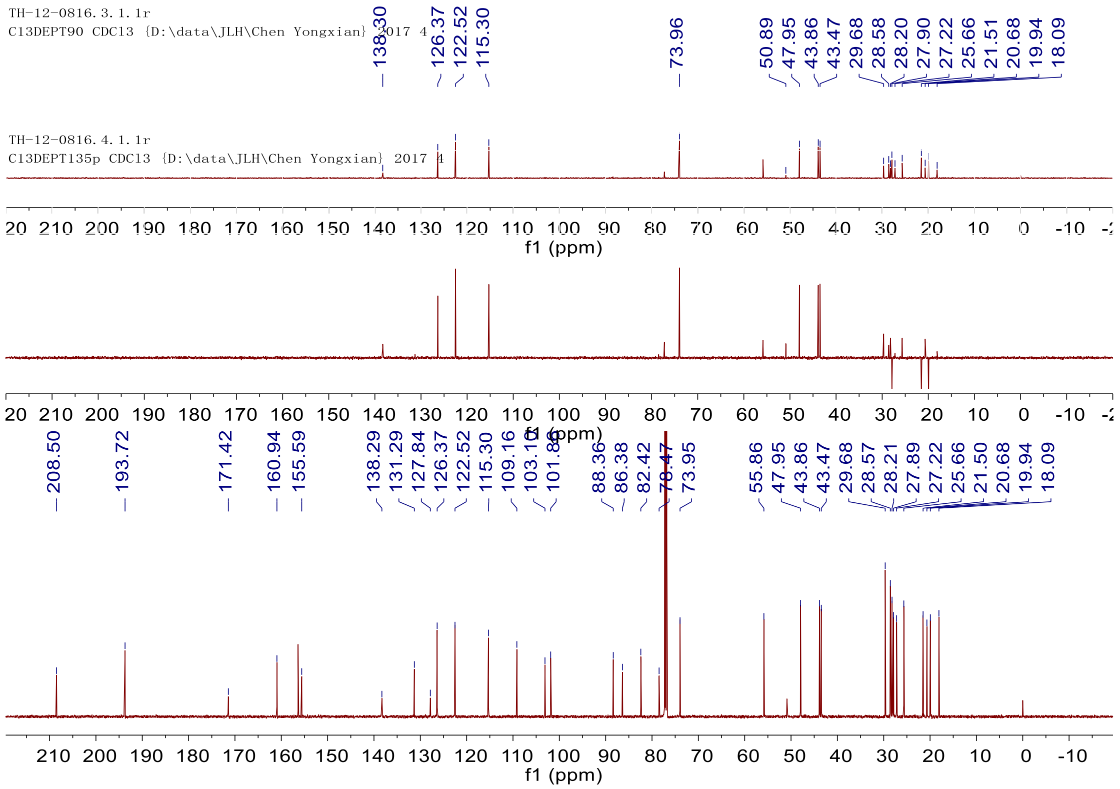

Figure S3. DEPT90 and DEPT135 of TH12-1

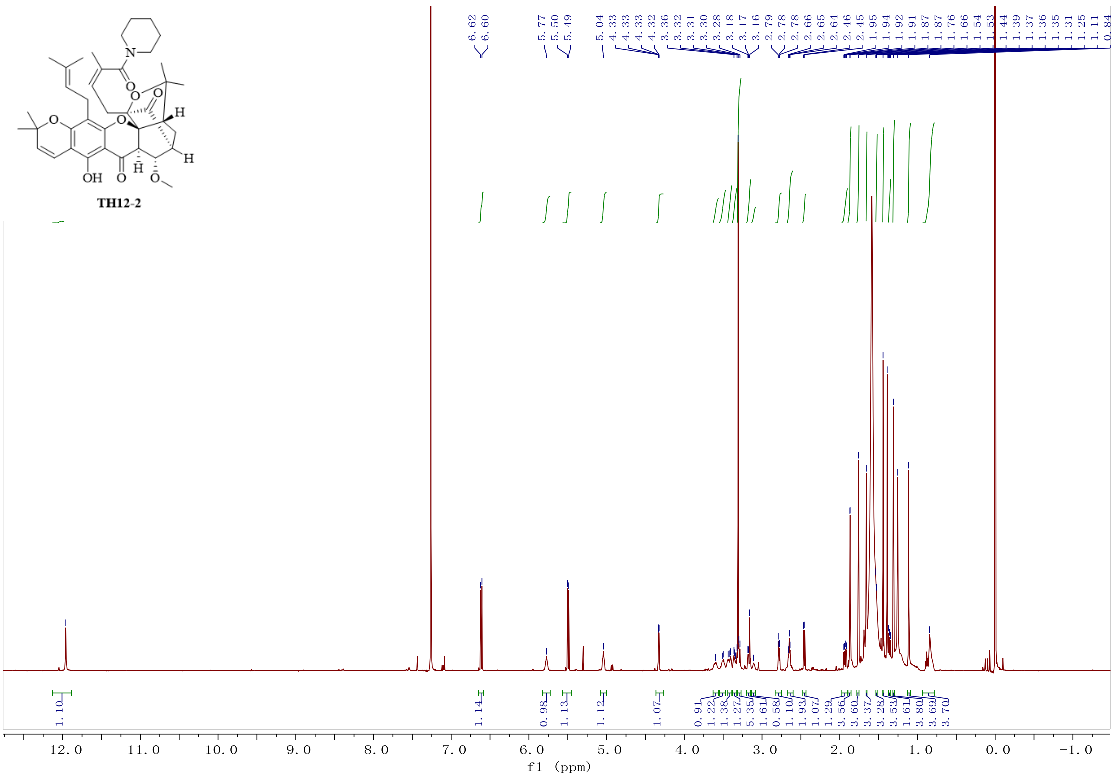

Figure S4. ^{1}H NMR of TH12-2

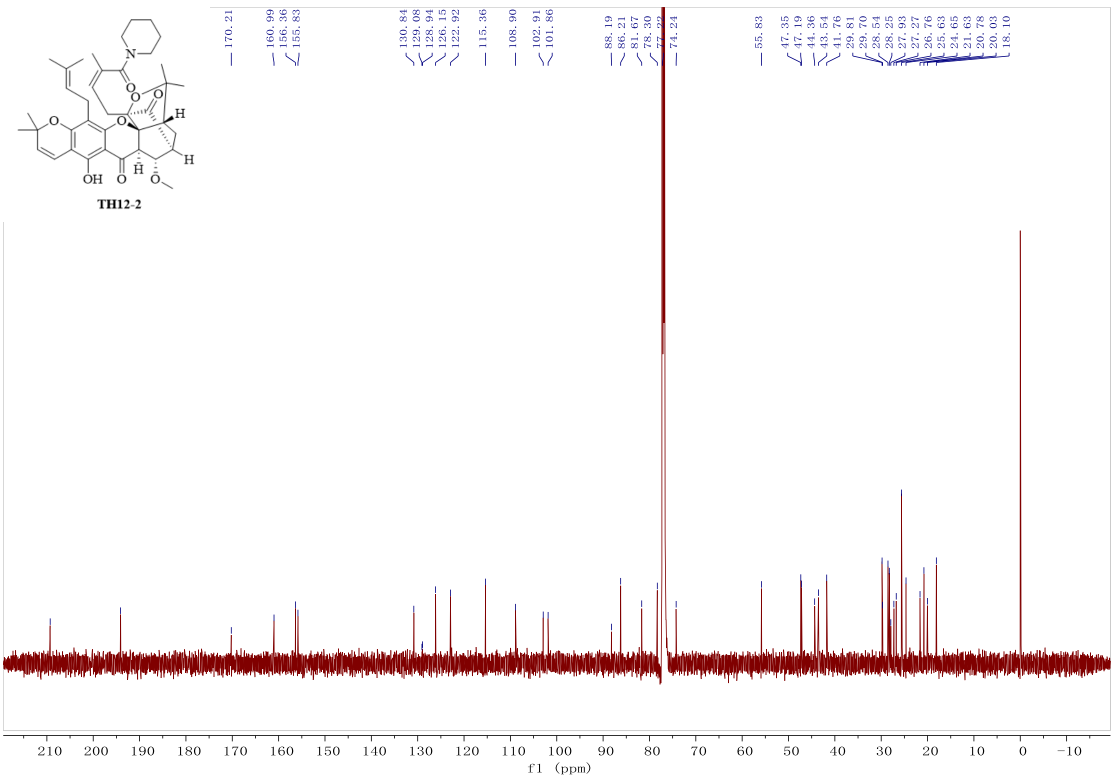

Figure S5. ^{13}C NMR of TH12-2

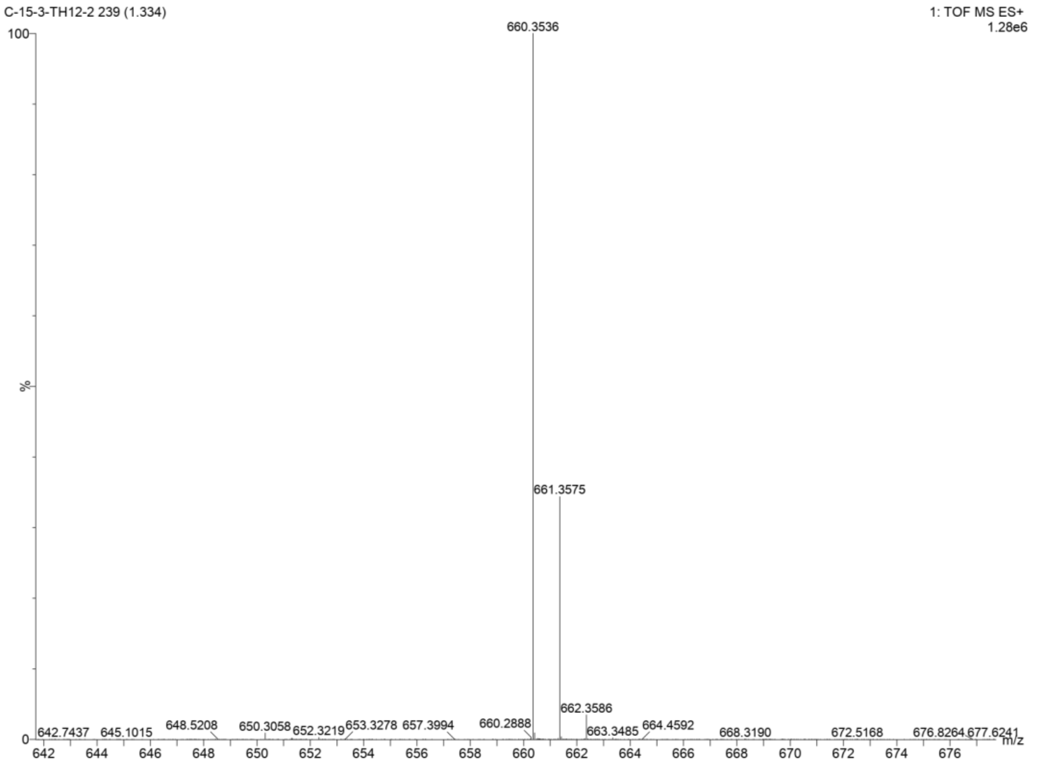

Mass	Calc. Mass	mDa	PPM	DBE	i-FIT	Norm	Conf(%)	Formula
660.3536	660.3536	0.0	0.0	15.5	590.3	n/a	n/a	C39 H50 N O8

Figure S6. HRMS of TH12-2

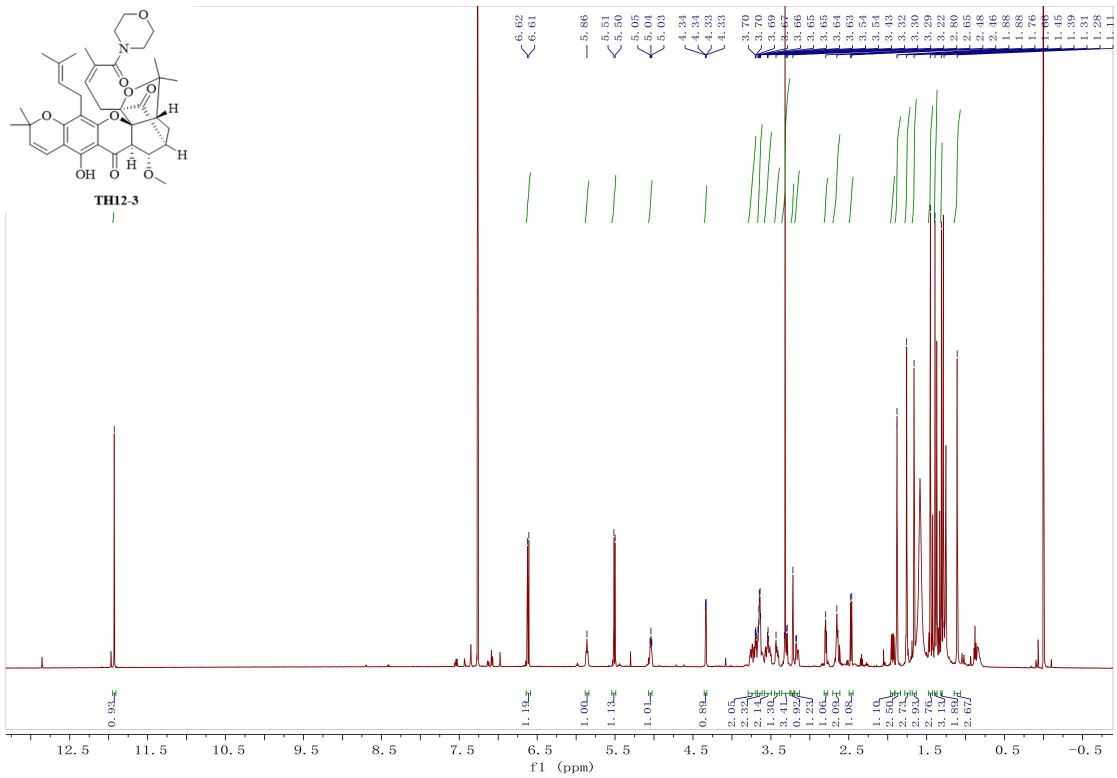

Figure S7. ^{1}H NMR of TH12-3

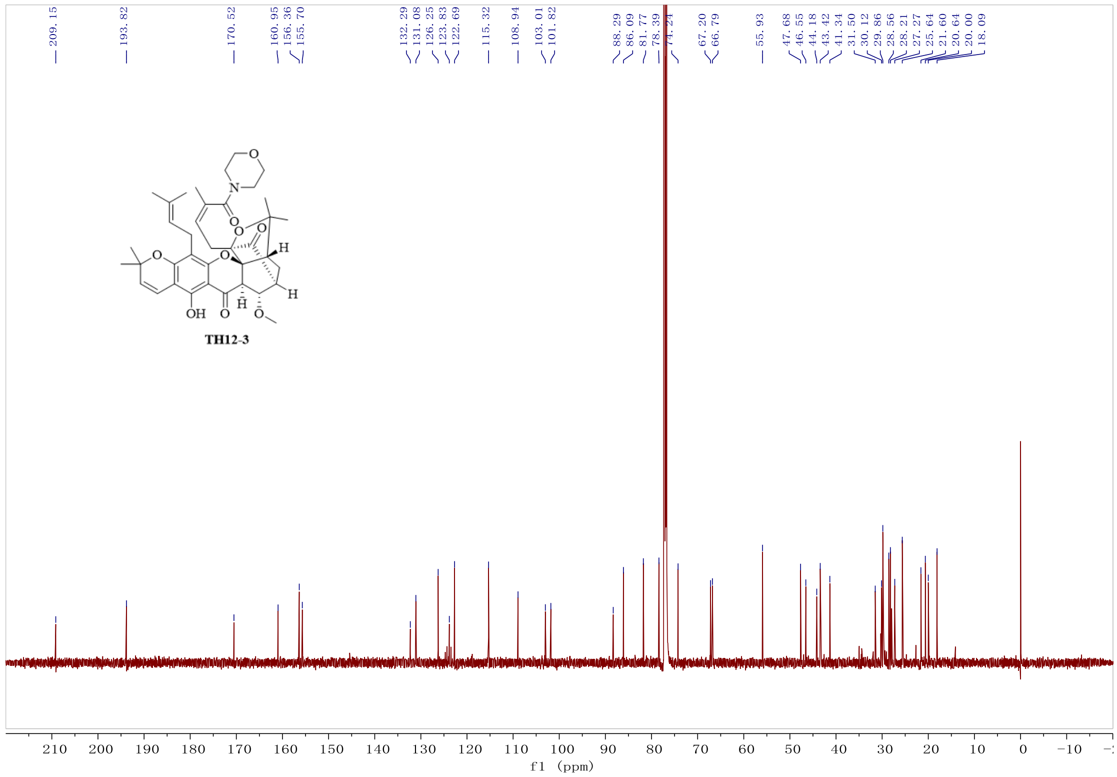

Figure S8. ^{13}C NMR of TH12-3

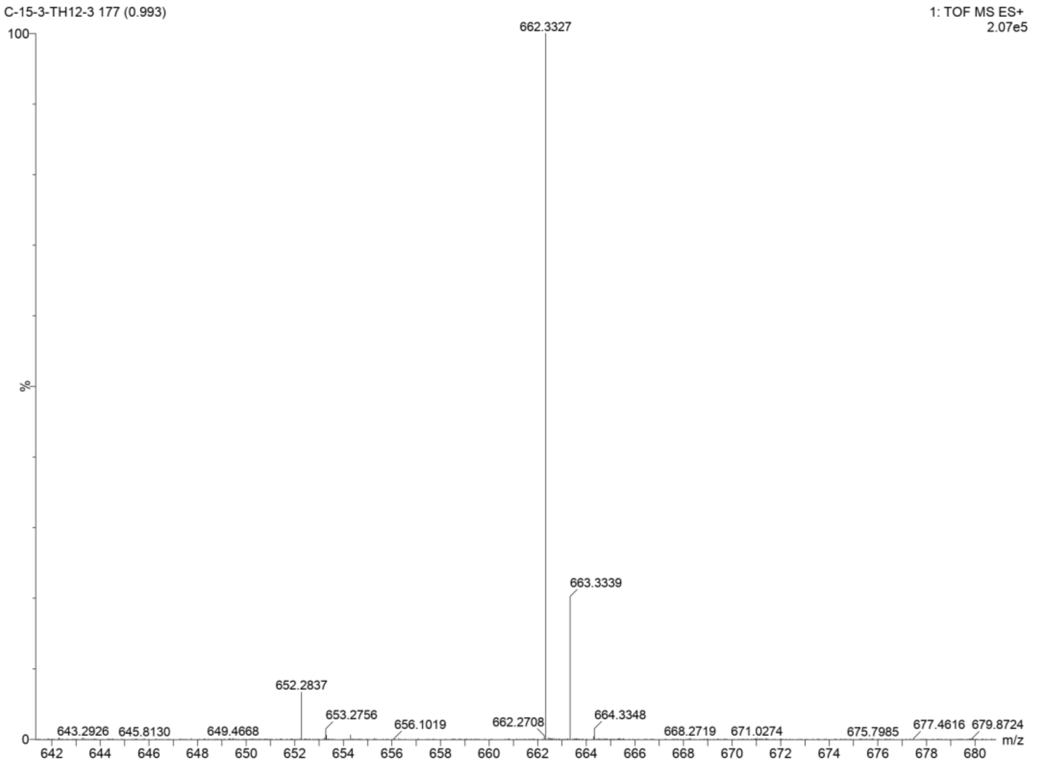

Mass	Calc. Mass	mDa	PPM	DBE	i-FIT	Norm	Conf(%)	Formula
662.3327	662.3329	-0.2	-0.3	15.5	393.6	n/a	n/a	C38 H48 N O9

Figure S9. HRMS of TH12-3

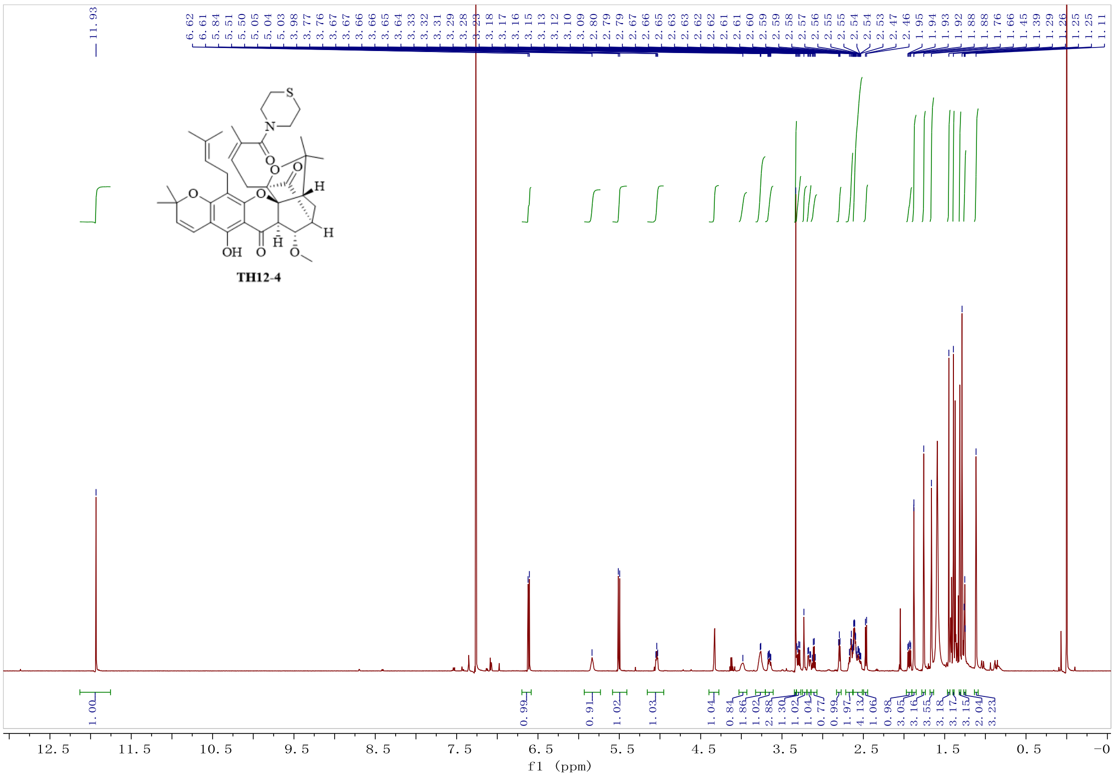

Figure S10. ^{1}HNMR of TH12-4

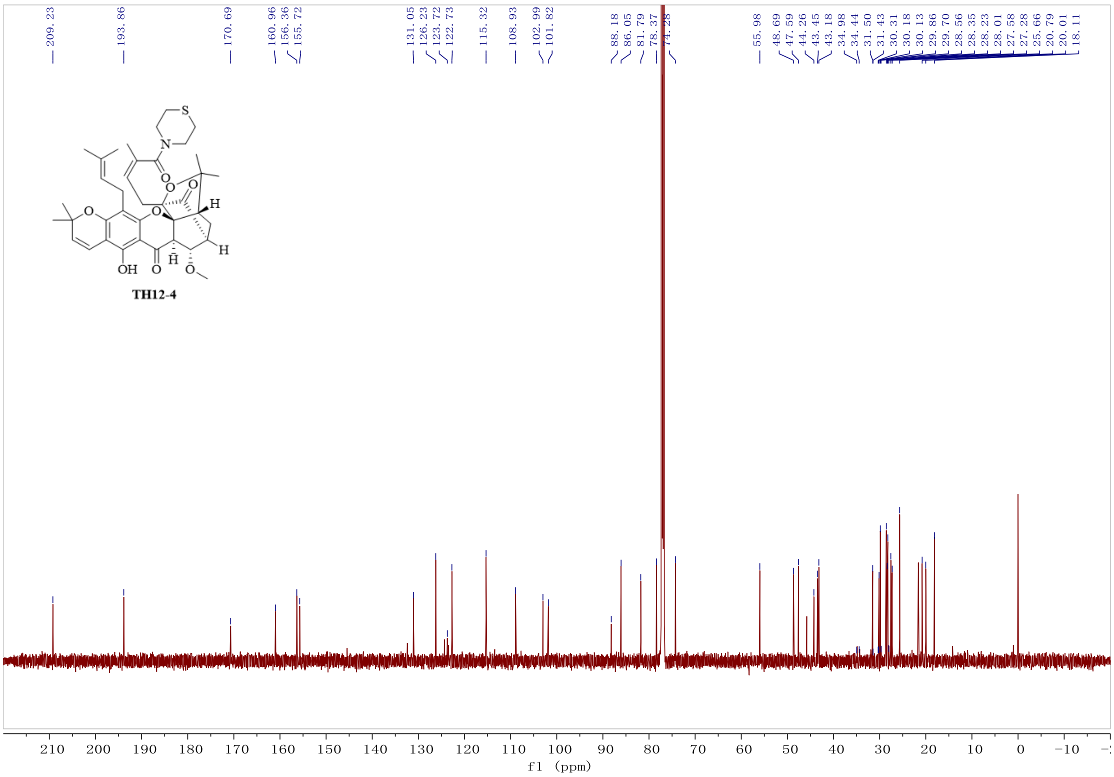

Figure S11. ^{13}C NMR of TH12-4

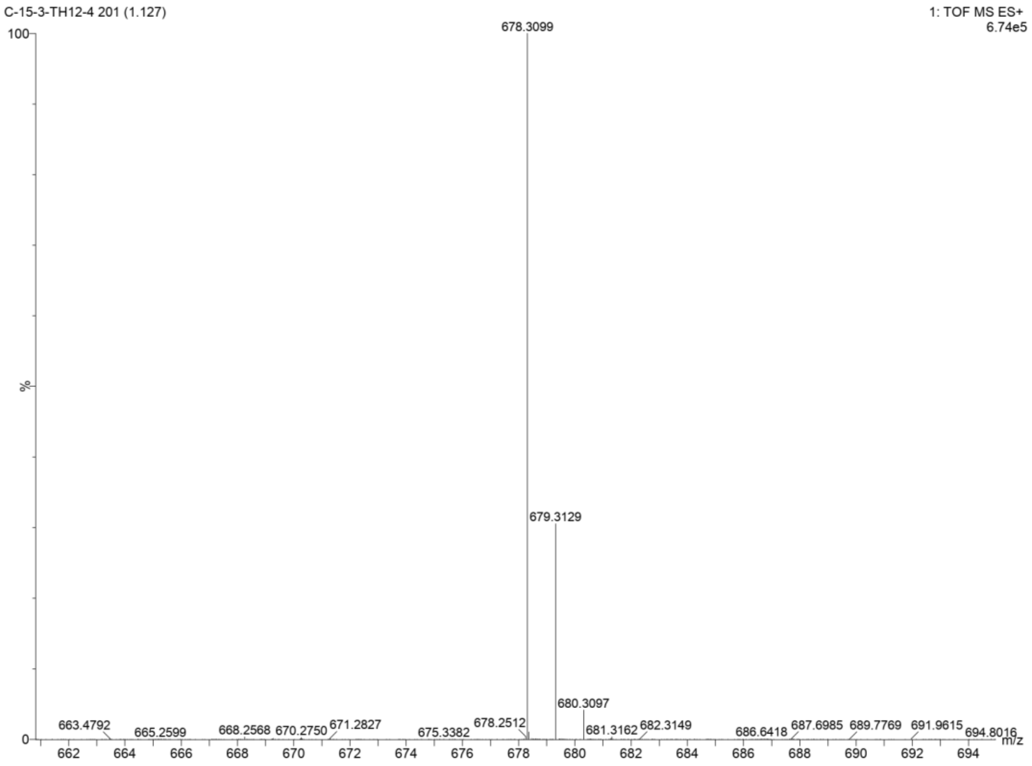

Mass	Calc. Mass	mDa	PPM	DBE	i-FIT	Norm	Conf(%)	Formula
678.3099	678.3101	-0.2	-0.3	15.5	446.4	n/a	n/a	C38 H48 N O8 S

Figure S12. HRMS of TH12-4

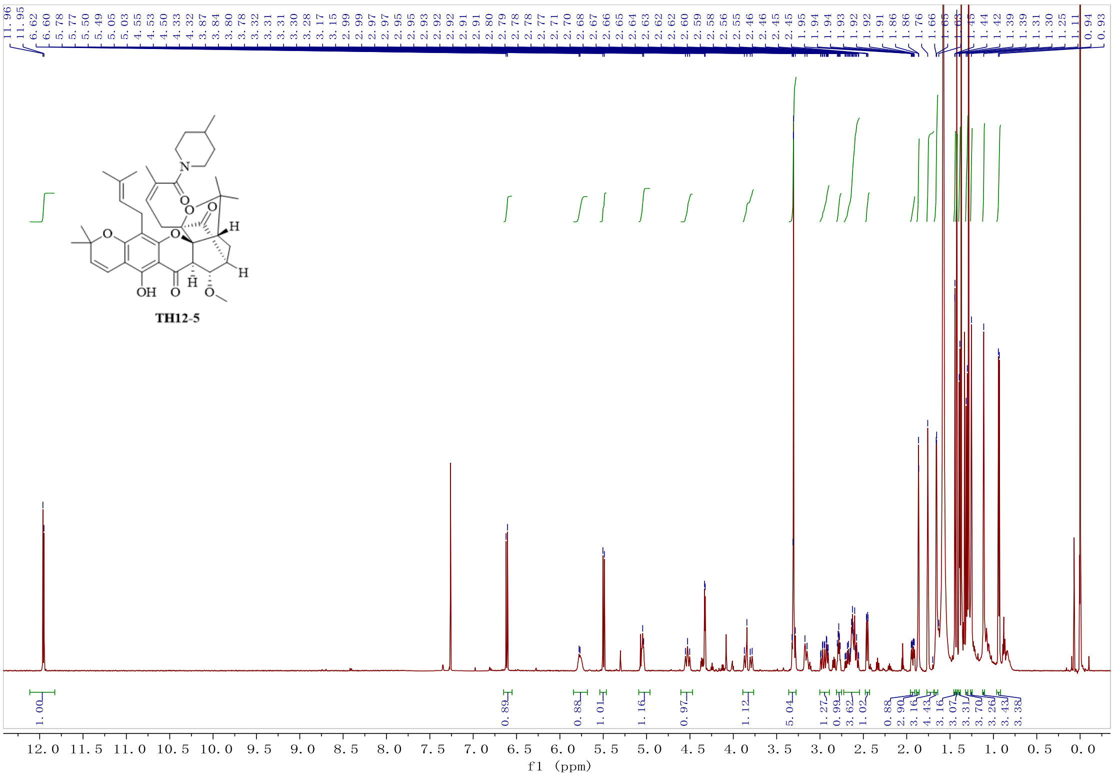

Figure S13. ^{1}HNMR of TH12-5

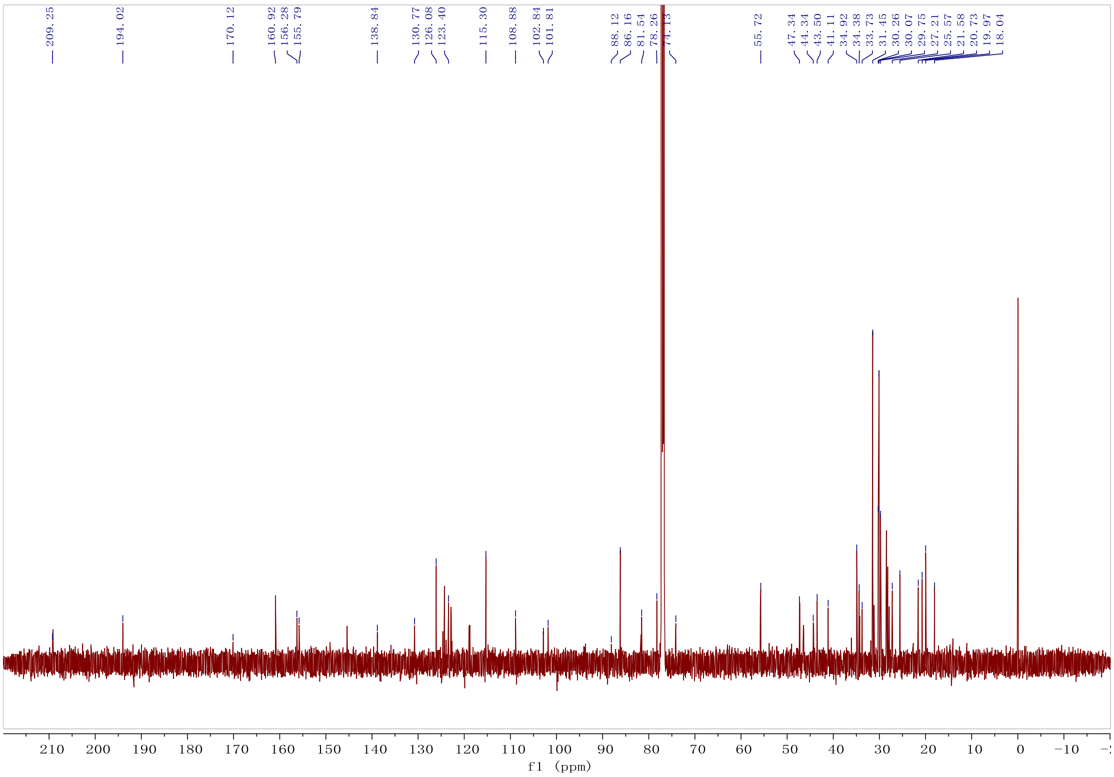

Figure S14. ^{13}C NMR of TH12-5

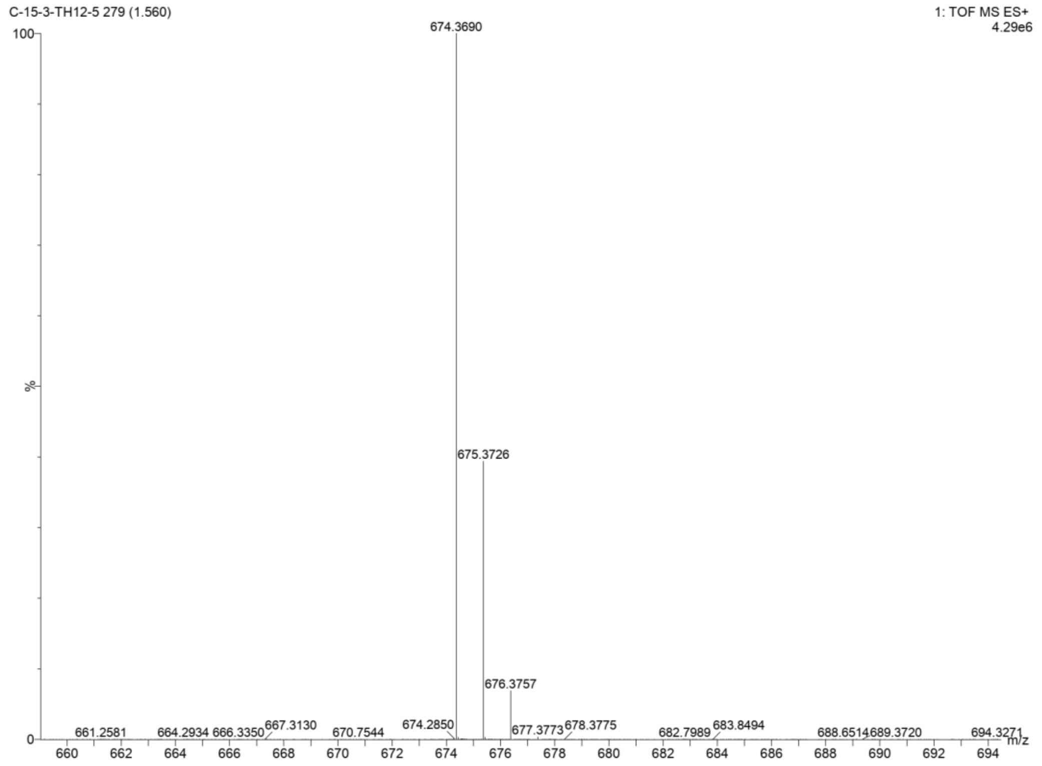

Mass	Calc. Mass	mDa	PPM	DBE	i-FIT	Norm	Conf(%)	Formula
674.3690	674.3693	-0.3	-0.4	15.5	707.5	n/a	n/a	C40 H52 N O8

Figure S15. HRMS of TH12-5

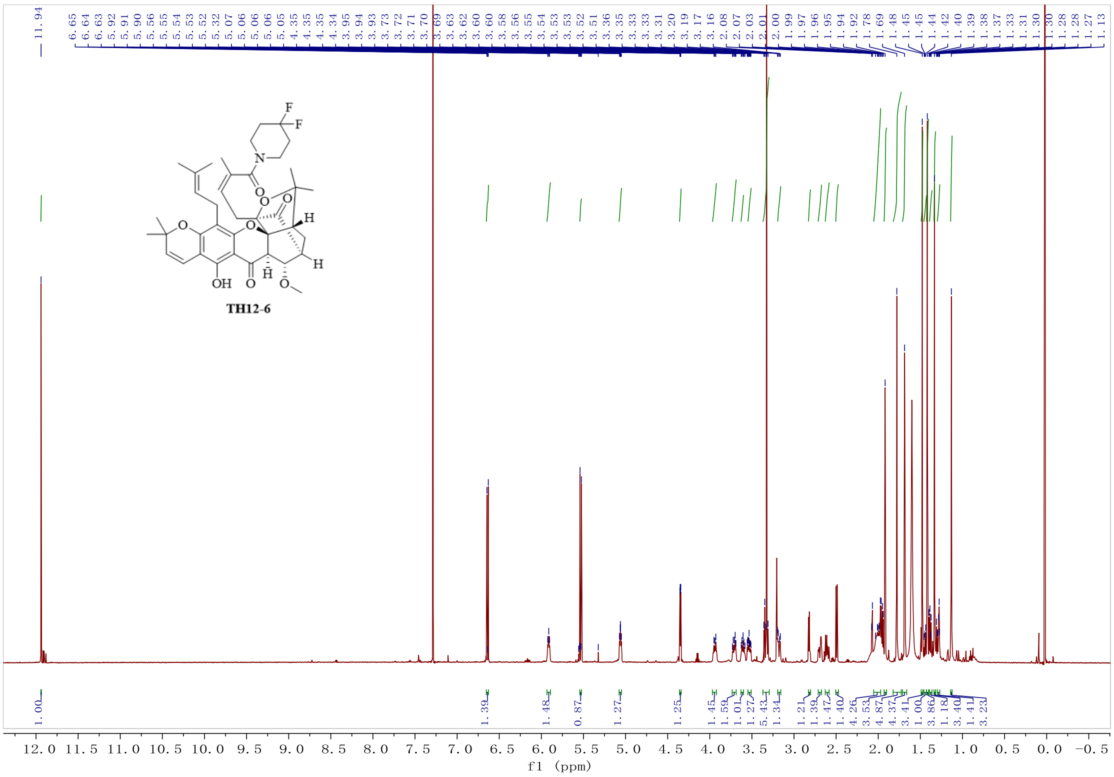

Figure S16. ^{1}H NMR of TH12-6

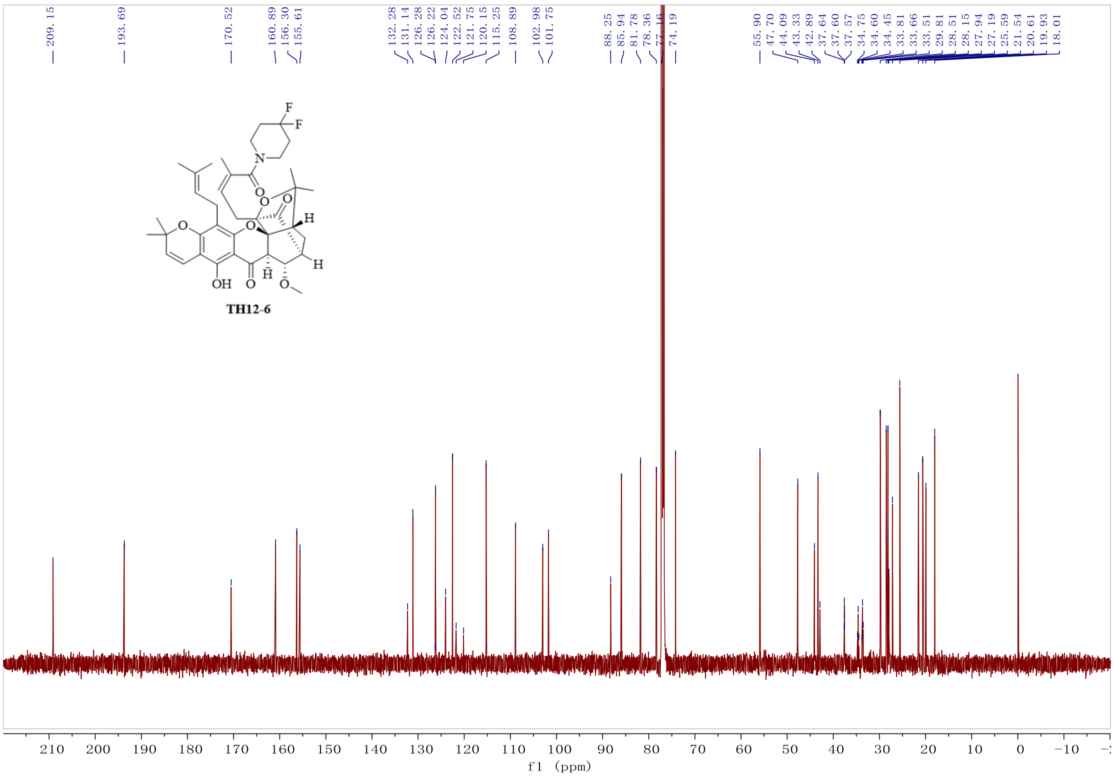

Figure S17. ^{13}C NMR of TH12-6

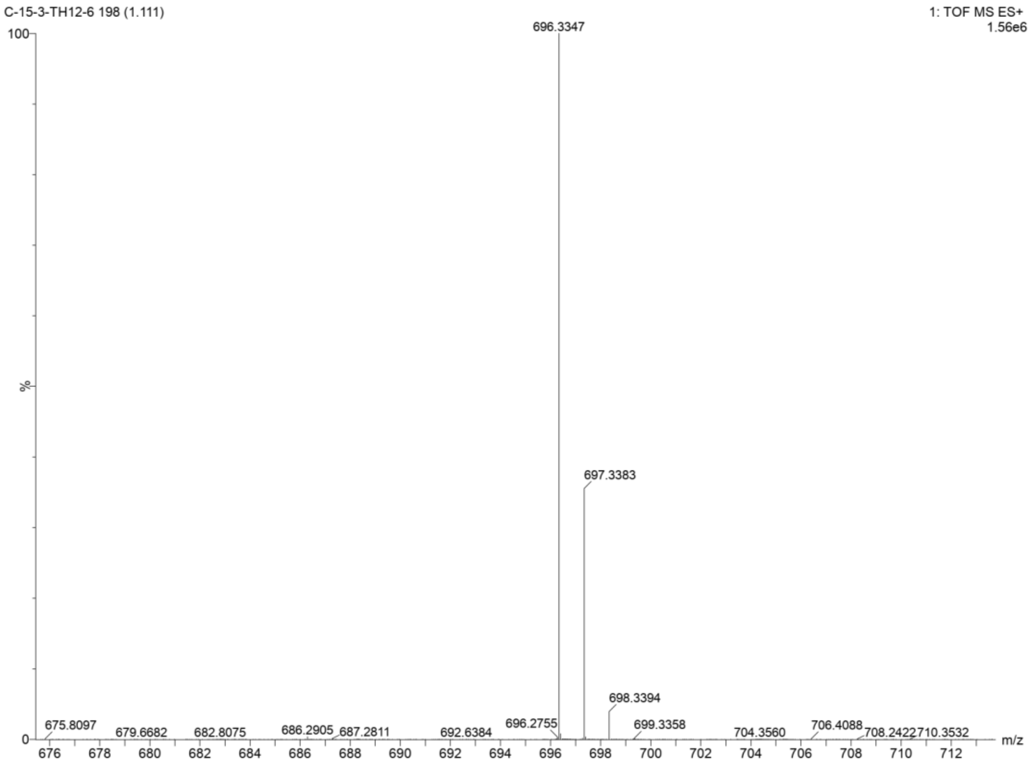

Mass	Calc. Mass	mDa	PPM	DBE	i-FIT	Norm	Conf(%)	Formula
696.3347	696.3348	-0.1	-0.1	15.5	580.7	n/a	n/a	C39 H48 N O8 F2

Figure S18. HRMS of TH12-6

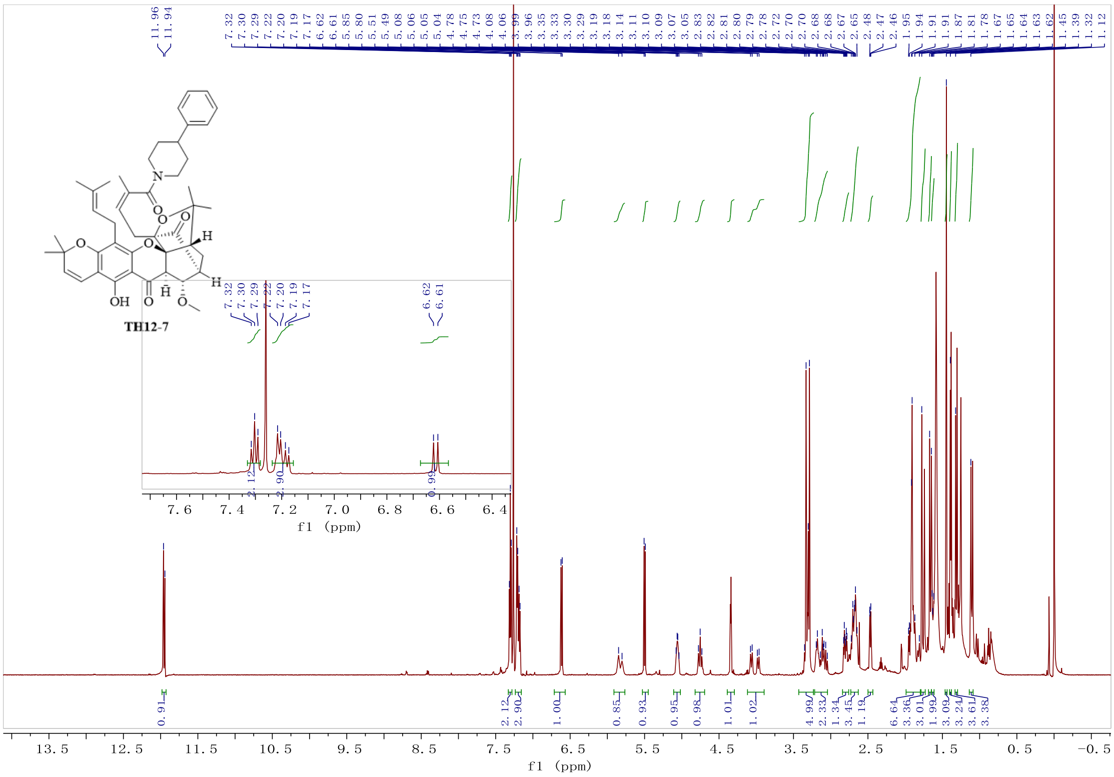

Figure S19. ^{1}H NMR of TH12-7

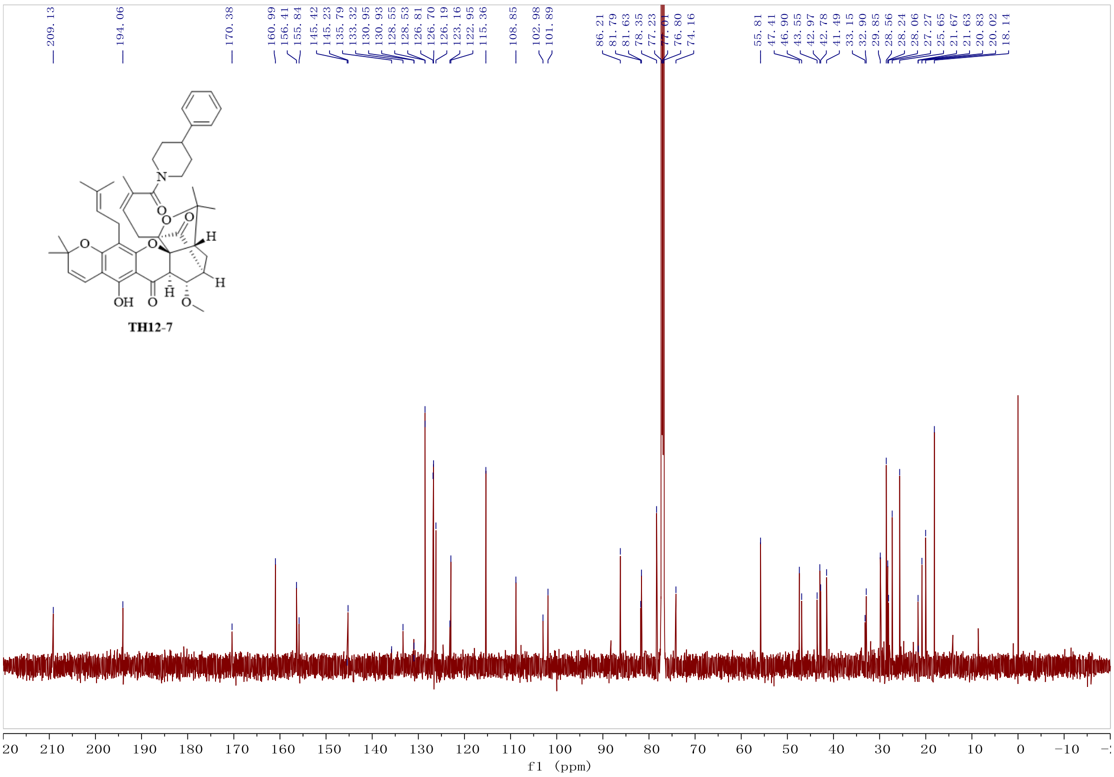

Figure S20. ^{13}C NMR of TH12-7

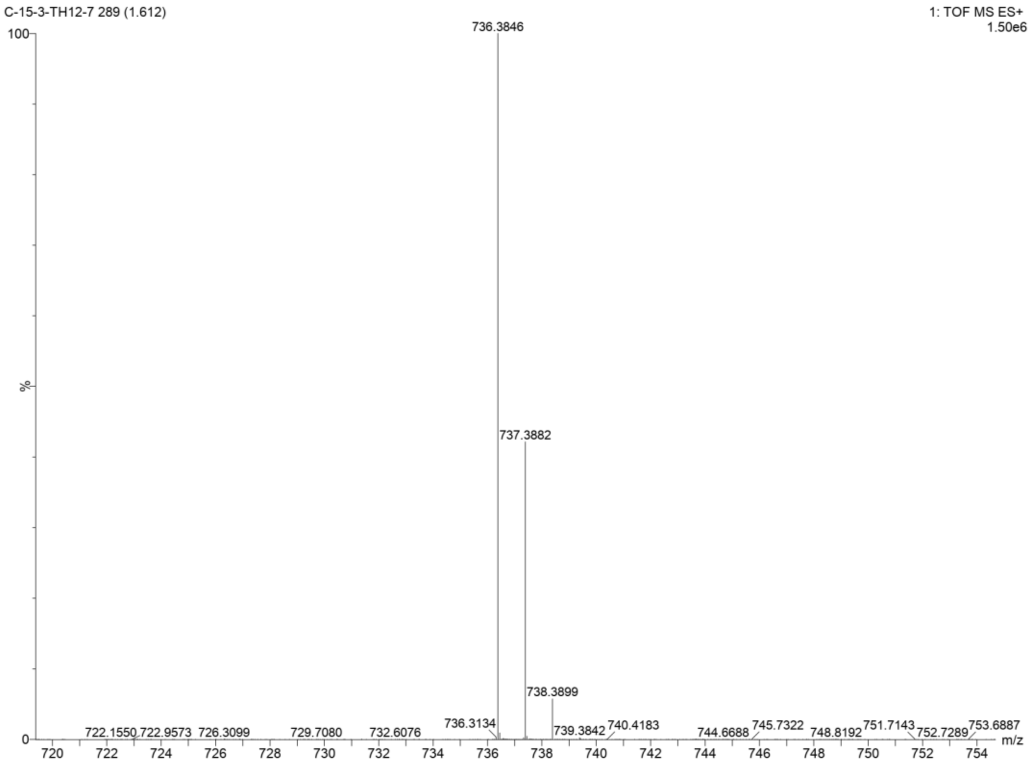

Mass	Calc. Mass	mDa	PPM	DBE	i-FIT	Norm	Conf(%)	Formula
736.3846	736.3849	-0.3	-0.4	19.5	584.7	n/a	n/a	C45 H54 N O8

Figure S21. HRMS of TH12-7

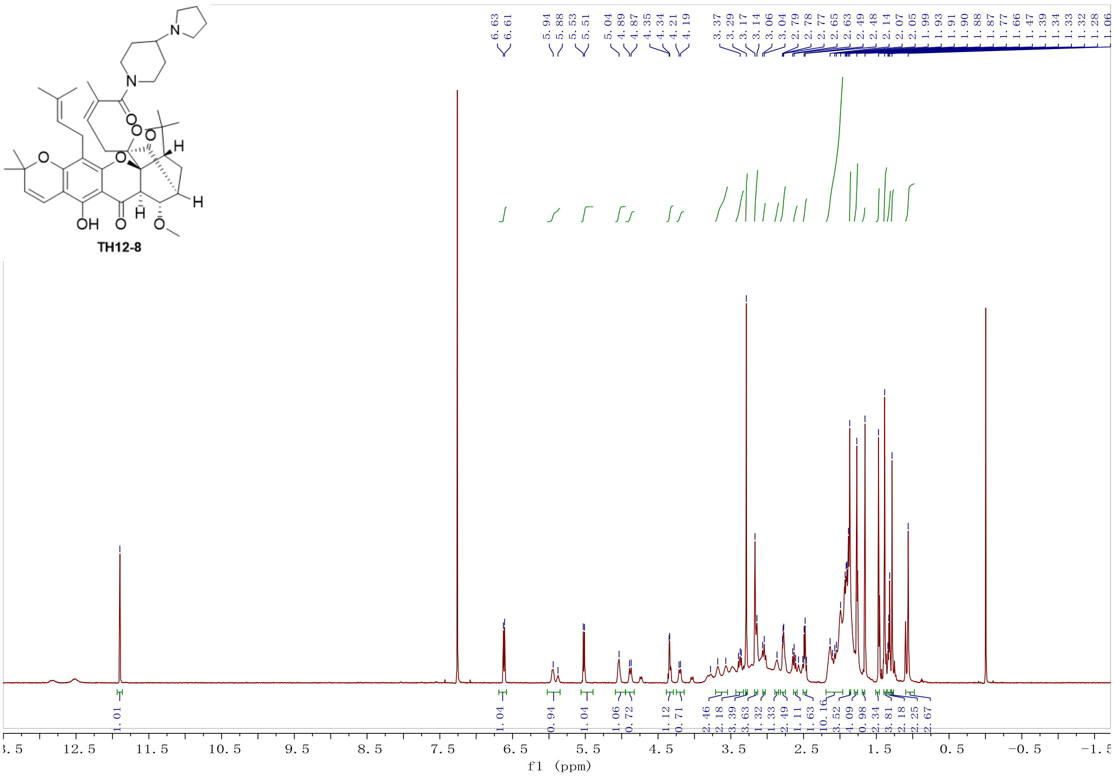

Figure S22. ^{1}H NMR of TH12-8

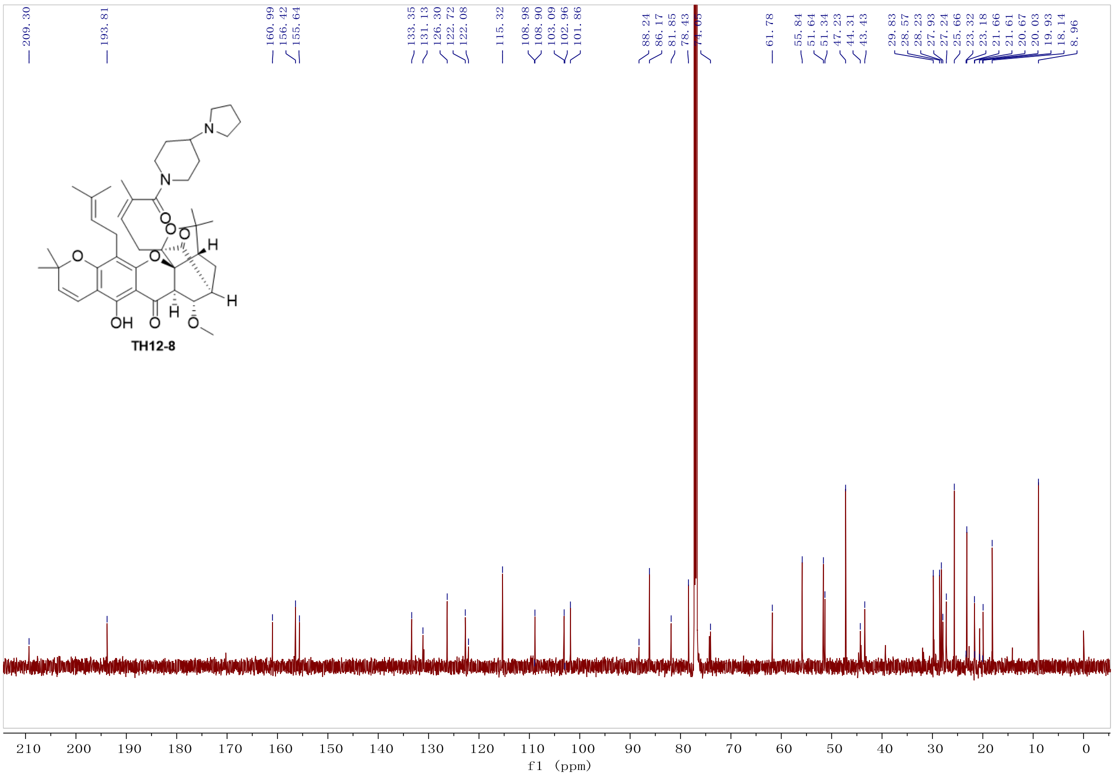

Figure S23. ^{13}C NMR of TH12-8

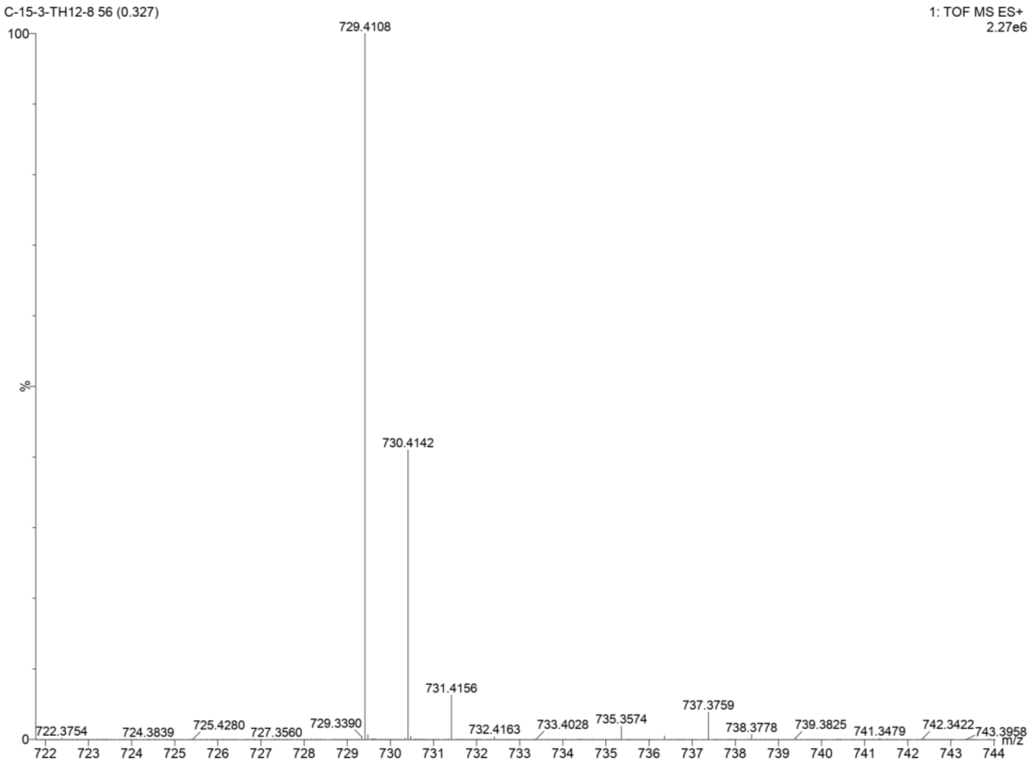

Figure S24. HRMS of TH12-8

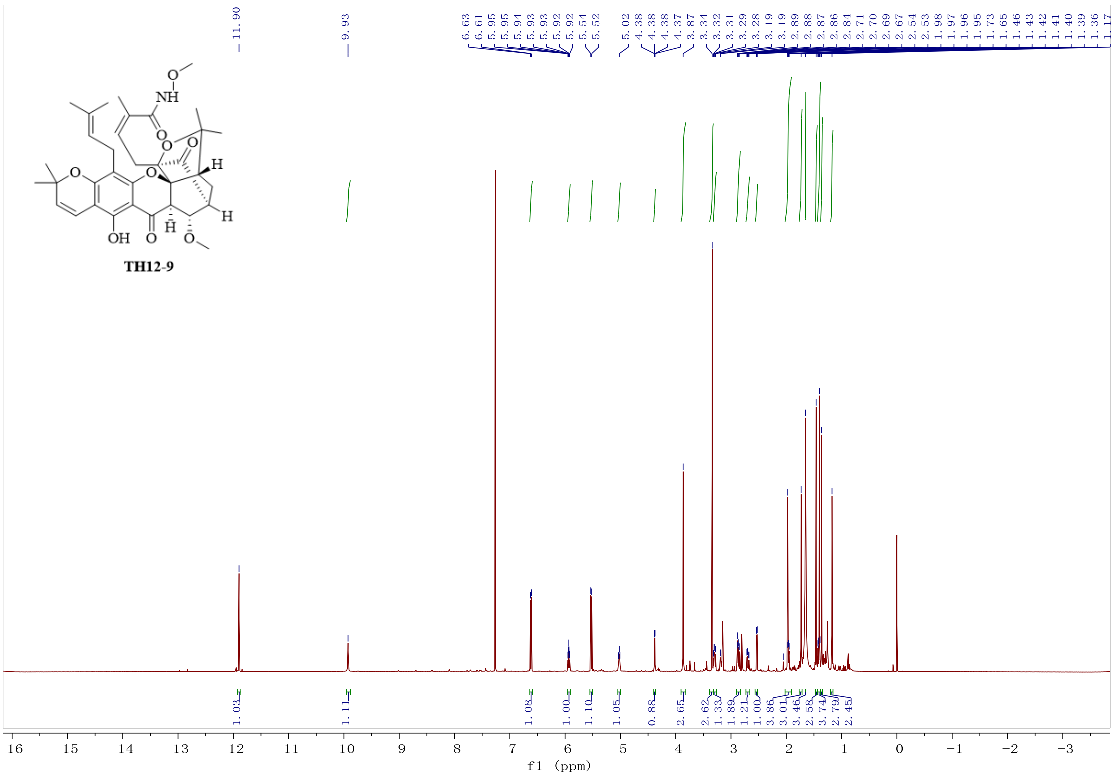

Figure S25. ^{1}H NMR of TH12-9

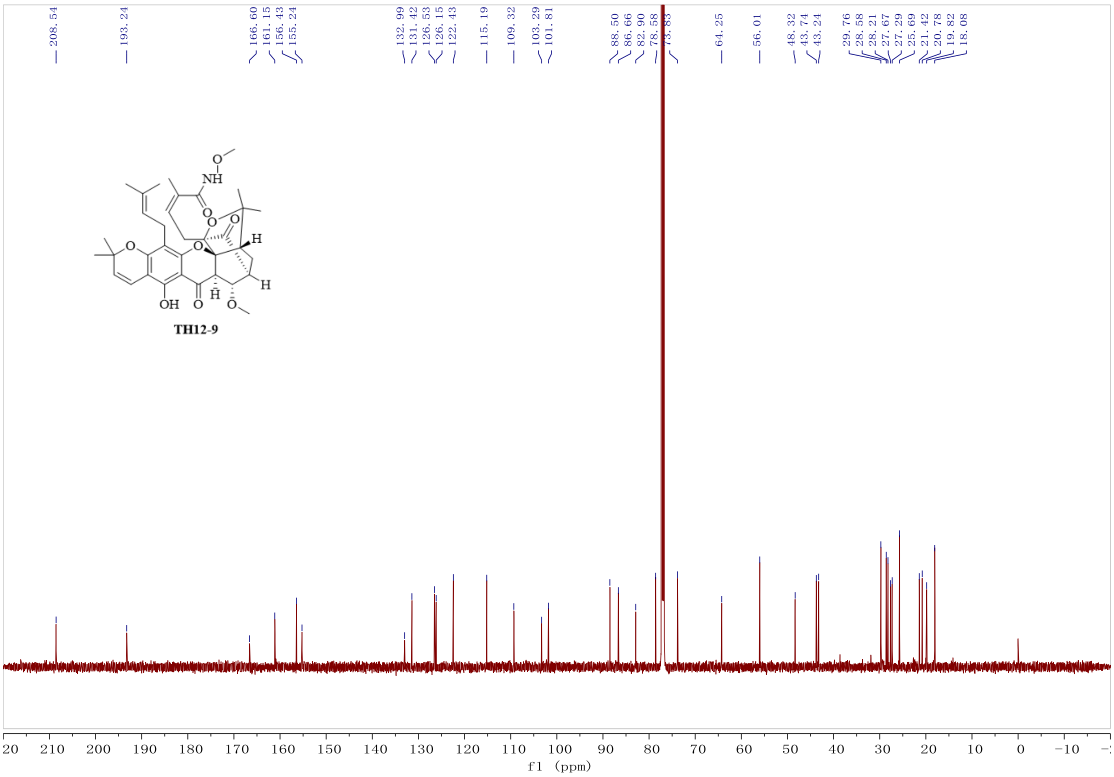

Figure S26. ^{13}C NMR of TH12-9

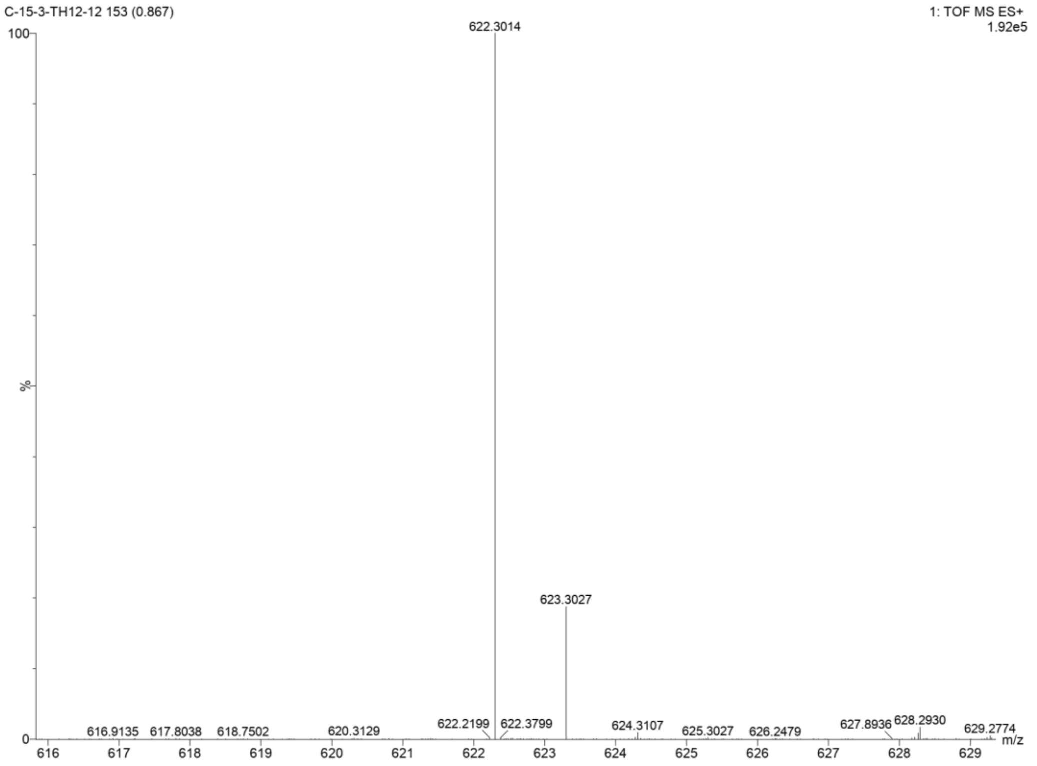

Mass	Calc. Mass	mDa	PPM	DBE	i-FIT	Norm	Conf(%)	Formula
622.3014	622.3016	-0.2	-0.3	14.5	367.1	n/a	n/a	C35 H44 N O9

Figure S27. HRMS of TH12-9

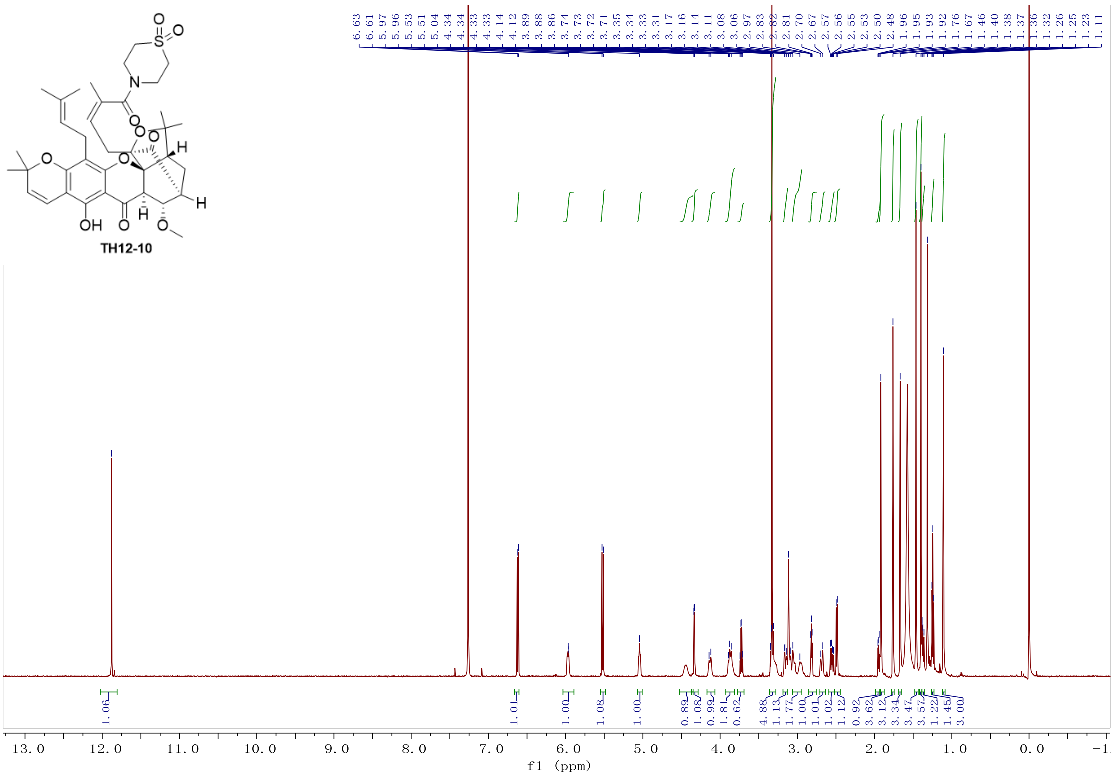

Figure S28. ^{1}H NMR of TH12-10

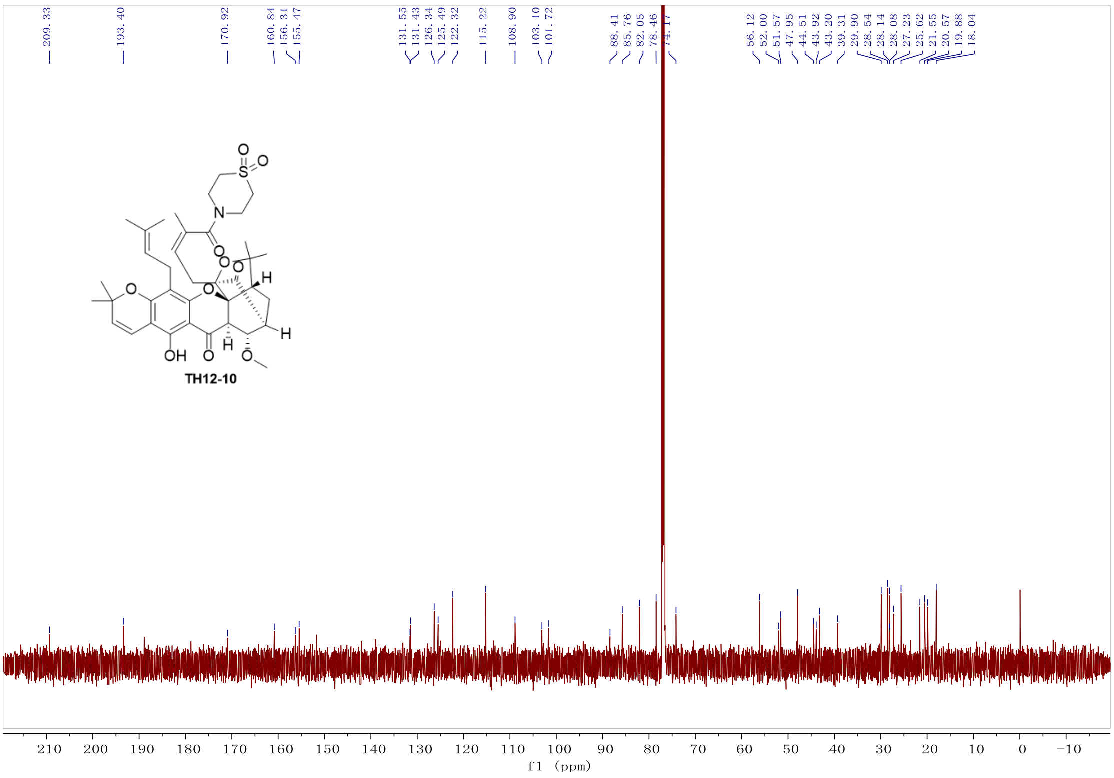

Figure S29. ^{13}C NMR of TH12-10

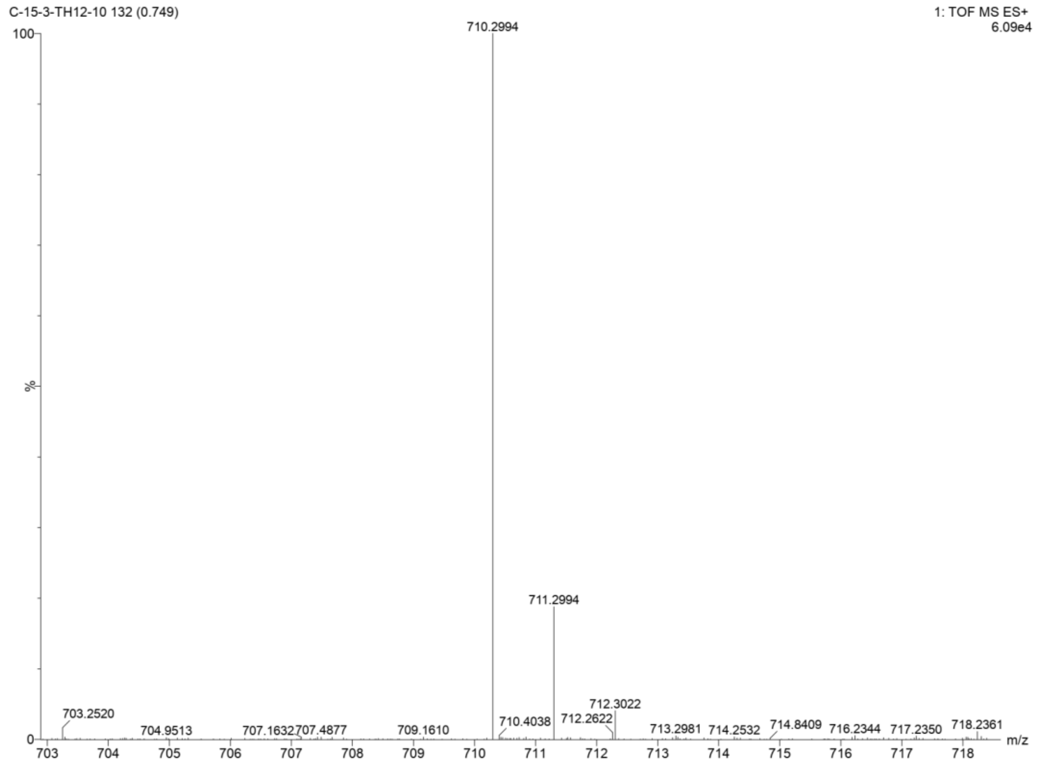

Mass	Calc. Mass	mDa	PPM	DBE	i-FIT	Norm	Conf(%)	Formula
710.2994	710.2999	-0.5	-0.7	15.5	311.5	n/a	n/a	C38 H48 N O10 S

Figure S30. HRMS of TH12-10
